# Mechanozyme: An Artificial Enzyme With a Mechanophore Framework

**DOI:** 10.1002/advs.75254

**Published:** 2026-04-20

**Authors:** Jiahao Ji, Pravin Pokhrel, Sajan Shakya, Bishal Pokhrel, Grinsun Sharma, Pratiksha Chaudhary, Hao Shen, Hanbin Mao

**Affiliations:** ^1^ Department of Chemistry and Biochemistry Kent State University Kent Ohio USA; ^2^ Advanced Materials and Liquid Crystals Institute Kent State University Kent Ohio USA; ^3^ School of Biomedical Sciences Kent State University Kent Ohio USA

**Keywords:** DNAzyme, marginal stability, mechanical modulation, mechanochemistry, ultrasonication

## Abstract

Conformational flexibility of natural enzymes, characterized by marginal stability, displays functional variations. In this work, we uncovered that artificial DNAzymes made of G‐quadruplex (GQ)‐hemin complexes also display this marginal stability phenomenon. Through single‐molecule fluorescent MT‐HILO (Magnetic Tweezers coupled with Highly Inclined and Laminated Optical sheet), we recorded the highest peroxidase activity among all known natural or artificial enzymes when the GQ mechanophore inside the DNAzymes was destabilized by an external force. We name enzymes with such force‐responsive marginal stability “mechanozymes” to reflect the mechanical modulation of enzymatic activities. To set the stage for mechanical modulation on mechanozymes beyond the single‐molecule level, ultrasonication was applied to enhance the catalytic function of a large ensemble of mechanozymes by weakening their GQ mechanophore structures. This work not only established marginal stability in artificial enzymes for the first time, but it also provided unprecedented mechanical modulation on catalytic activities, both of which are expected to have profound ramifications for catalysis exploited across the fields of chemistry and biosciences.

## Graphical abstract

Mechanical destabilization of a G‐quadruplex mechanophore in a DNAzyme markedly boosts its catalytic activity, demonstrating “mechanozymes” as artificial enzymes whose functions are modulated by mechanical stress exerted by ultrasonication. This mechanozyme principle is broadly applicable to control biomolecular activities.

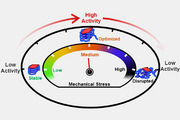

## 1 Introduction

In nature, the regulation of protein and enzyme activities through marginal stability is common [1, 2]. For instance, cold‐adapted enzymes such as psychrophilic citrate synthases exhibit reduced structural stability but enhanced catalytic activity at low temperatures [1]. In evolutionary biology, it is recognized that stability and performance of a protein are often inversely correlated [3]. To modulate enzymatic activity, various approaches—such as ligands [4] and buffer systems [5]—have been used to alter an enzyme's stability. For example, allosteric inhibitors can strengthen flexible regions of an enzyme, restricting molecular motion and thereby decreasing catalytic efficiency [6]. Similarly, binding of metal ions like Zn^2^^+^ can tune enzymatic activities [5] by limiting conformational flexibilities in enzymes. The marginal stability has been attributed to the flexible conformation in a destabilized catalyst, which facilitates the accommodation of the transient state of a molecule in a reaction. As a result, the activation energy is reduced, and catalytic efficiency is improved for the reaction [7].

Natural enzymes possess complex structures, with their active sites comprising only a small fraction of the overall framework. Such a property complicates efforts to modulate enzymatic activities since entire structures need to be considered. In contrast, synthetic enzymes like nanozymes [8, 9], DNAzymes [10, 11, 12], and coronazymes [13, 14] feature simpler structures, enabling easier structural modifications to tune for activities. A key question is whether these simpler structures exhibit marginal stability properties. Confirming such behavior could unlock innovative approaches to designing synthetic enzymes with significantly enhanced performance. While some studies have explored the stability of synthetic enzymes, there is no prior report that has systematically correlated stabilities to activities [14, 15, 16, 17, 18, 19].

The stability of proteins or artificial enzymes can be tuned by mutations or environmental factors. Genetic mutations may alter overall topologies, which, in turn, affect activities. This feature makes it difficult to nail down the marginal stability principle. Environmental factors such as temperature [20] and chemical denaturants [21, 22] can alter structural stability. However, they also face the implication that different environmental settings can alter catalytic activities by themselves. A rarely explored universal environmental factor is mechanical force. By applying an external force, almost all proteins or artificial enzymes can reversibly change their structural stabilities while other environmental conditions, such as temperature and solvent compositions, kept constant. Therefore, the measured activity of enzymes under external force truly reflects the effect of varied structural stability. Beyond biological enzymes, mechanical stress has also been reported to modulate catalytic activity in the heterogeneous catalysis system [23, 24]. In industrial processes, mechanical stress can either enhance or suppress enzymatic activity [25, 26], yet the determinants of these divergent effects remain unclear. Without a predictive relationship between structural stability and catalytic efficiency under mechanical force, rational design of force‐responsive catalytic systems remains challenging. Functional groups such as mechanophores are known to change their structural properties in response to mechanical force [27, 28, 29]. Here, we propose that for mechanophores with enzymatic activities, they can serve as synthetic enzymes themselves. As activities of these synthetic enzymes can be controlled by mechanical force, we conceptualize such mechanophore‐based artificial enzymes as mechanozymes.

In this work, we demonstrated such a mechanozyme, a DNAzyme made of G‐quadruplex (GQ)‐hemin complex. In this complex, hemin functions as a redox catalytic center around which a GQ serves as a mechanophore skeleton with force‐responsive properties [27, 30]. After investigating the correlation between structural stability and catalytic activity among various GQ‐hemin DNAzymes, we found that the GQ‐hemin mechanozymes with lower stabilities exhibited higher catalytic activities. This confirmed the marginal stability principle in artificial enzymes for the first time. Using MT‐HILO (Magnetic Tweezers coupled with Highly Inclined and Laminated Optical sheet) at the single‐molecule level, we revealed that mechanical force increased the activity of the mechanozyme by weakening the stability of the artificial enzyme structure. To address the low throughput involved in the single‐molecule setting, we applied ultrasonication to a bulk solution containing a massive set of mechanozymes. Again, we found that sonication‐induced mechanical force compromises the stability of the GQ mechanophore, leading to increased mechanozyme activities surpassing known natural and artificial peroxidases. Since mechanical force is a universal factor to vary structural stabilities, which, in turn, change enzymatic activities via the marginal stability principle, we anticipated that the mechanozymes demonstrated here represent a generic concept widely applicable in fields such as catalysis, therapeutics, and biosensing.

## 2 Results and Discussion

### 2.1 Anticorrelation Between Activity and Stability in an Ensemble Set of Artificial Enzymes

**FIGURE 1:**
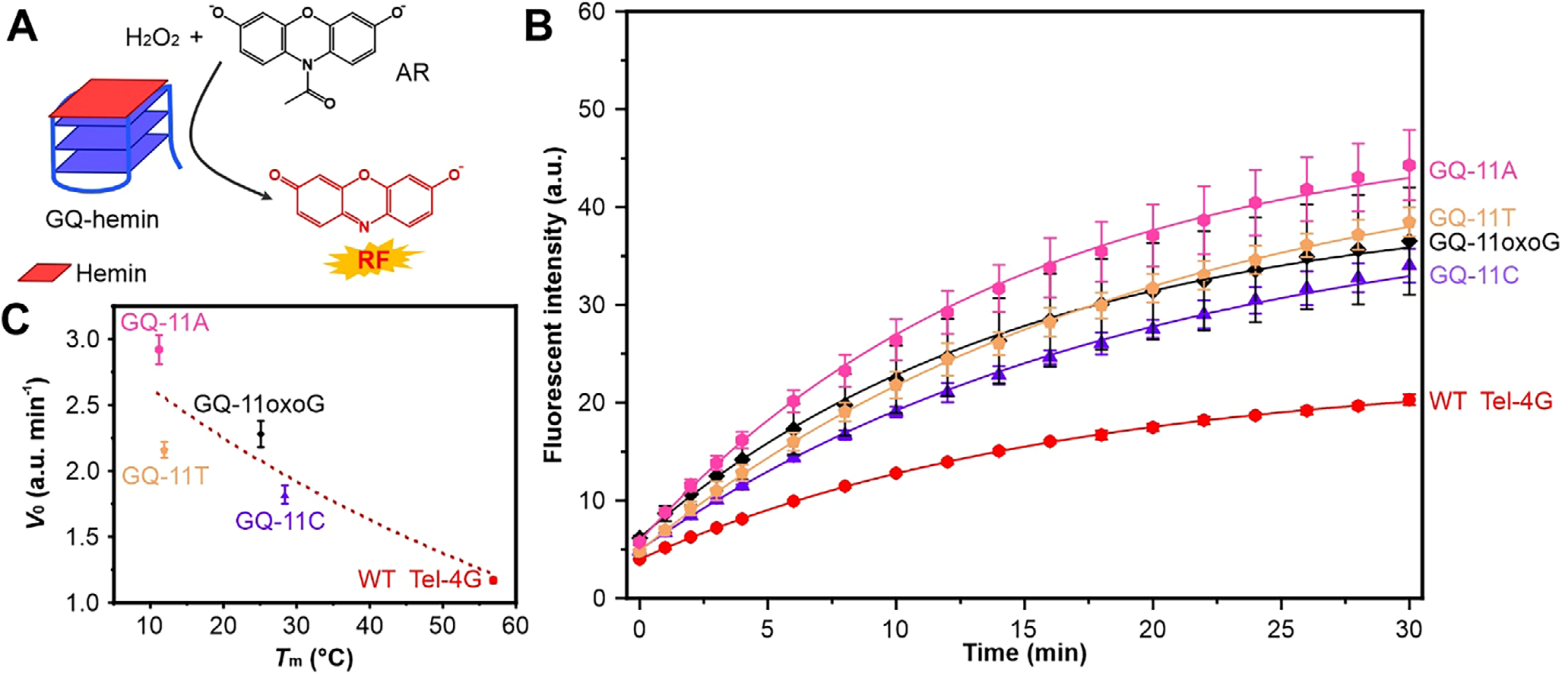
Ensemble‐averaged activities of DNAzymes consisting of a hemin bound to different GQ sequences. (A) Schematic of GQ‐hemin catalysis. (B) Ensemble‐averaged RF fluorescence increases over time, catalyzed by different DNAzymes in a buffer of 0.5 mm h_2_ o_2_, 10 µm AR, 40% (w/v) sucrose, 100 mm KCl, 10 mm Tris at pH 7.4. (C) Initial velocity (*V*_0_) versus melting temperature of DNAzymes (*T*_m_) (details see Section S4). The data in (C) were fitted by a single‐exponential equation to guide the eyes (dotted red lines). Error bars depict standard deviations from *n* = 3 independent measurements. The fluorogenic reactions were performed at 25°C.

To investigate the relationship between catalytic activity and stability of artificial enzymes in a bulk solution, we used G‐quadruplex (GQ)‐hemin, a peroxidase‐mimicking DNAzyme that converts Amplex Red (AR), a non‐fluorescent substrate, into resorufin (RF), a fluorescent product, in the presence of hydrogen peroxide (H_2_O_2_) (Figure 1A). We designed human telomere GQ mutants with varying stabilities by replacing the ^11^G in the wild‐type human DNA Tel‐4G sequence (5′‐TTA GGG TTA ^10^G^11^G^12^G TTA GGG TTA GGG TTA‐3’) with adenine (A), cytosine (C), thymine (T) or 8‐oxoguanine (oxoG). The resulting variants were named GQ‐11A, GQ‐11C, GQ‐11T or GQ‐11oxoG, respectively (Figure 1A; Table S1). As the mutant residue is located in the middle G‐tetrad plane, this design minimizes the effect of flanking sequences on the mutant DNAzyme structure and activity. CD (circular dichroism) showed either hybrid or parallel GQ conformations for these mutants (Figure S1). Previous studies have shown that hybrid and parallel GQ conformations demonstrate similar catalytic activities when they are complexed with hemin molecules [31, 32].

We expect these GQ mutants to have different thermal stabilities, which allows us to evaluate the catalytic reactivity of DNAzymes from the perspective of structural stabilities. To confirm this, the stabilities of these GQ mutants were measured by UV melting experiments [33]. Indeed, we found that the wild‐type human telomere GQ (WT Tel‐4G) had the highest melting temperature (*T*_m_ = 56.9 ± 0.6°C), followed by GQ‐11C (28.4 ± 0.3°C), GQ‐11oxoG (25.1 ± 0.1°C), GQ‐11T (11.9 ± 0.1°C), and GQ‐11A (11.2 ± 0.5°C) (details see Sections S1 and S3 and Figure S2).

Next, we measured the catalytic activities of these GQ‐hemins. To this end, we formed the GQ‐hemin complex by incubating each GQ sequence (25 µm) with hemin (25 µm) at a 1:1 ratio in a sucrose buffer (40% (w/v) sucrose, 100 mm KCl, 10 mm Tris at pH 7.4). The DNAzyme (0.25 µm) thus prepared was then mixed with 0.5 mm H_2_O_2_ and 10 µm AR (details see Section S4 and Figures S3 and S4). Using a fluorescence microscope, we measured the initial velocity (*V*_0_) catalyzed by each GQ‐hemin [32]. Among four GQ mutant‐hemin complexes, the GQ‐11A had the highest *V*_0_ (2.92 ± 0.11 a.u. min^−1^, Table S2), followed by GQ‐11oxoG (2.28 ± 0.10 a.u. min^−1^), GQ‐11T (2.16 ± 0.06 a.u. min^−1^), GQ‐11C (1.82 ± 0.07 a.u. min^−1^), and WT Tel‐4G GQ (1.17 ± 0.02 a.u. min^−1^). When we plotted the *V*_0_ vs. *T*_m_ of the DNAzymes (Figure 1C), we found that the higher the DNAzyme activities, the lower the stabilities, exhibiting a strong negative correlation (Pearson correlation coefficient *r* = −0.96). This result is consistent with the marginal stability principle demonstrated in natural enzymes.

### 2.2 Confirmation of the Marginal Stability Principle in DNAzymes at the Single Molecular Level

**FIGURE 2:**
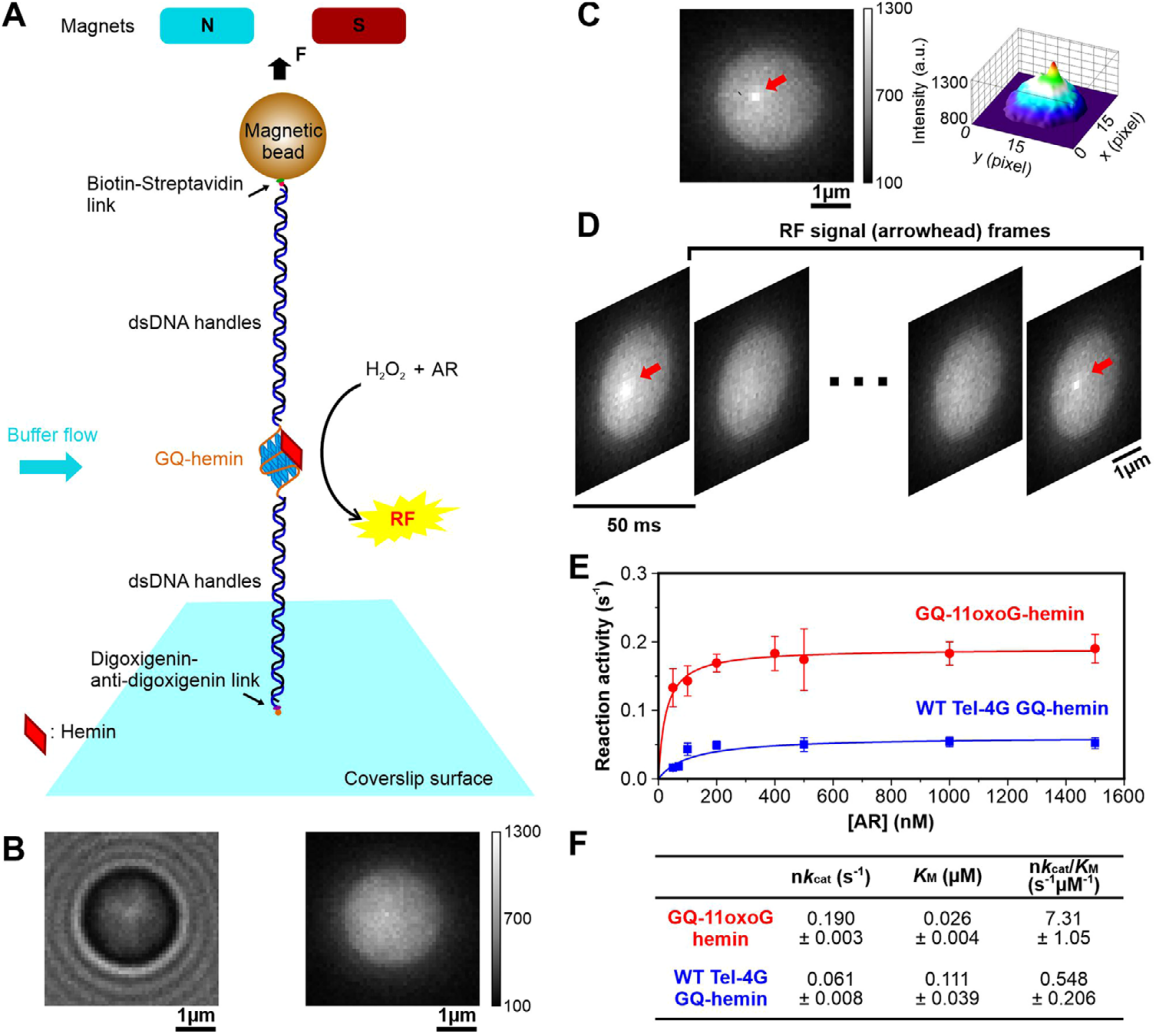
Single‐molecule fluorescence experiments to measure catalytic activities of individual GQ‐hemin DNAzymes (WT Tel‐4G GQ‐hemin or GQ‐11oxoG‐hemin). (A) Schematic of the single‐molecule fluorescence MT‐HILO setup. GQ‐hemin complexes were immobilized between the coverslip surface and a superparamagnetic bead via two double‐stranded DNA handles (see Sections S1, S5, and S6 and Figures S5–S8 for details). (B) Representative bright‐field (left) and fluorescence (right) images showing a single GQ‐hemin‐tethered magnetic bead. (C) A typical fluorescence signal (indicated by an arrowhead) from the resorufin (RF) product catalyzed by a single GQ‐hemin complex (left). The corresponding 3D fluorescence intensity histogram is shown to the right. Each image pixel corresponds to a 108 nm × 108 nm area. (D) Temporal image frames with single‐molecular RF signals catalyzed by a single GQ‐hemin complex. The exposure time for each frame was 50 ms. (E) Dependence of the catalytic activity of individual GQ‐hemin complexes (WT Tel‐4G‐hemin or GQ‐11oxoG‐hemin) on the concentration of AR, measured under 3 mW 532 nm linear laser excitation in the presence of 3 mm H_2_O_2_. Data were fitted using a modified Michaelis–Menten kinetic model (see text). (F) Comparison of the catalytic efficiencies of WT Tel‐4G‐hemin and GQ‐11oxoG‐hemin complexes. Error bars represent standard deviations derived from at least 10 independent measurements. Reactions were conducted at 25°C in sucrose buffer (40% (w/v) sucrose, 100 mm KCl, 10 mm Tris, pH 7.4) with 3 mm H_2_O_2_ and varying concentrations of AR (50–1500 nm) under 3 mW 532 nm laser excitation.

To confirm that lower stability is correlated with higher activity in DNAzymes, we performed highly sensitive single‐molecule assays using magnetic tweezers coupled with a highly inclined and laminated optical sheet (MT‐HILO) [13, 34]. To evaluate catalytic activities of DNAzymes, the WT Tel‐4G or GQ‐11oxoG sequence was ligated to two double‐stranded DNA (dsDNA) handles (see Section S5 and Figure S5 for details). One handle was immobilized on the coverslip surface via a digoxigenin–anti‐digoxigenin interaction, while the other was tethered to a superparamagnetic bead through a biotin–streptavidin linkage (Figure 2A; Figure S6). A pair of magnets generated a magnetic field that exerted a controllable tensile force on the DNA construct through the superparamagnetic bead, with the magnitude of the force modulated by adjusting the distance between the magnets and the coverslip.

During experiments, a small tensile force of 1.0 pN was maintained in the DNA construct, which ensured that the GQ‐hemin complex was levitated, thereby minimizing surface effects while facilitating mass transport. For catalytic measurements, 3 mm hydrogen peroxide (H_2_O_2_) with varying concentrations (50–1500 nm) of AR were introduced into the microfluidic chamber (Figure S7) in the same sucrose buffer used above (40% (w/v) sucrose, 100 mm KCl, 10 mm Tris, pH 7.4). As depicted in Figure 1A, the GQ‐hemin converted non‐fluorescent AR into fluorescent resorufin (RF), which was observed using the MT‐HILO microscope to calculate the reaction rate constants of single GQ‐hemin complexes (Figure 2B–D).

We found that the rate of the reaction (*v*) catalyzed by the WT Tel‐4G GQ‐hemin increases with AR concentration until a plateau is reached (Figure 2E blue). According to the modified Michaelis–Menten kinetics [14]:

(1) $$\upsilon =nk_{\mathrm{cat}}\left[S\right]/\left(K_{M}+\left[S\right]\right)$$

where *k*_cat_ is the reaction rate constant associated with a single catalytic site, *n* is the number of the reactive site in one enzyme, *K*_M_ is the Michaelis–Menten constant, and [*S*] is the concentration of the substrate. We found that *nk*_cat_ and *K*_M_ are 0.061 ± 0.008 s^−1^ and 0.111 ± 0.039 µm, respectively, for the WT Tel‐4G GQ‐hemin complex (Figure 2F). These two values are 0.190 ± 0.003 s^−1^ and 0.026 ± 0.004 µm, respectively, for the GQ‐11oxoG‐hemin (Figure 2F). When we calculated catalytic efficiency, *nk*_cat_/*K*_M_, we found that GQ‐11oxoG‐hemin (7.31 ± 1.05 s^−1^µm ^−1^) has 13 times higher efficiency than that of WT Tel‐4G GQ‐hemin (0.548 ± 0.206 s^−1^µm ^−1^). In fact, it is one of the highest values among current DNAzymes with peroxidase‐like activities [14, 35, 36]. As the overall stability of GQ‐11oxoG‐hemin (*T*_m_ = 11.5 ± 6.4°C and the weighted unfolding force average = 6.6 pN, see Figure S14, Table S4, and Section S7 under the subheading “Calculation of weighted unfolding force average” for details) is much weaker than WT Tel‐4G GQ‐hemin (*T*_m_ = 59.9 ± 0.5°C and the weighted unfolding force average = 22.9 pN), the higher catalytic activity in the GQ‐11oxoG DNAzyme observed here confirmed the marginal stability principle in DNAzymes.

### 2.3 Mechanical Modulation of Catalytic Activities in Individual DNAzymes

**FIGURE 3:**
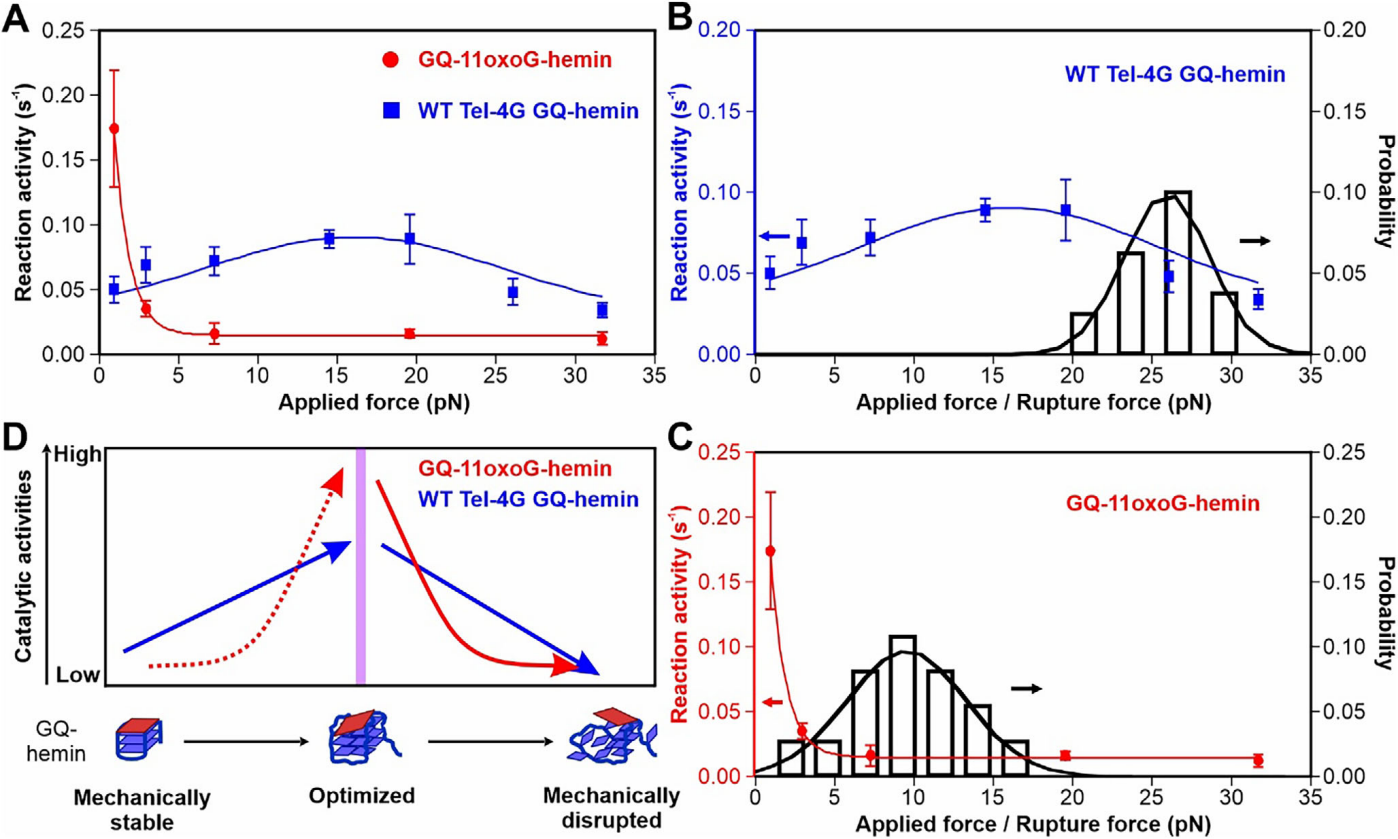
Effect of force on single‐molecule GQ‐hemin catalysis. (A) Catalytic activities of WT Tel‐4G and GQ‐11oxoG‐hemin under different forces. Solid curves were fitted by Gaussian equation (blue) or single‐exponential equation (red) to guide the eyes. (B) Rupture force histogram of the WT Tel‐4G GQ‐hemin (right axis). As a comparison, the activity of the WT Tel‐4G GQ‐hemin versus applied force is also shown (left axis). (C) Rupture force histogram of the GQ‐11oxoG‐hemin (right axis). As a comparison, the activity of the GQ‐11oxoG‐hemin versus applied force is also shown (left axis). (D) Schematic diagram showing the relationship between catalytic activity and stability of DNAzymes. Note that catalytic activity increases with stability until a maximum value is reached, beyond which the catalytic activity decreases with mechanical stability due to unfolded GQ structures at higher force levels. The dotted red arrow depicts a hypothetical case for GQ‐11‐oxoG‐hemin below 1 pN, which could not be measured reliably. Error bars depict the standard deviations from at least 7 catalysts. The reactions were performed at 25°C with 3 mm h_2_ o_2_ and 0.5 µm AR in a sucrose buffer (40% (w/v) sucrose, 100 mm KCl, 10 mm Tris at pH 7.4). These GQ‐hemins were exposed to 3 mW 532 nm linear laser light during the experiments.

Given that mechanical force can destabilize chemical structures such as those involved in DNAzymes [16, 37, 38], next, we interrogated DNAzyme activities when each enzyme was subject to different mechanical forces using the MT‐HILO setup (Figure 2A; Figure S8, see details in Section S6). With increasing tensile force applied to the WT Tel‐4G GQ‐hemin complex, we observed that reaction rate constants were increased from 0.050 ± 0.010 s^−1^ at 1.0 pN to 0.089 ± 0.007 s^−1^ at 14.5 pN (Figure 3A blue). At higher forces, however, the reaction rate constants were decreased from 0.089 ± 0.019 s^−1^ at 19.5 pN to 0.034 ± 0.006 s^−1^ at 31.6 pN (Figure 3A, blue). At low force regimes (<20 pN), increasing mechanical force is expected to have better catalytic activity for the GQ‐hemin DNAzyme due to decreased structural stability of the DNAzyme. However, higher forces (20–31.6 pN) may compromise the GQ structures in such a way that hemin may no longer bind, decreasing the catalytic activities. This result is consistent with the rupture force histogram (see Section S7 under the subheading “Optical tweezers results”, Figures S9–S13 and Tables S3–S5), which showed the structural stability of WT Tel‐4G GQ‐hemin around 26 pN, with the onset of structural disruption occurring around 20 pN (Figure S15).

In contrast, the catalytic activity of the GQ‐11oxoG‐hemin complex consistently decreased with increasing force (Figure 3A, red). As discussed in Figure S16, the average mechanical stability of the GQ‐11oxoG‐hemin complex was around 10 pN, while the complex started to unfold as low as 2–3 pN. This indicates that when the applied force was increased from 1.0 to 3.8 pN, the structural integrity of the GQ‐11oxoG‐hemin complex could be compromised in such a way that the catalytic reaction was much reduced, as hemin may not be able to bind to the GQ‐11oxoG, which was indeed observed in Figure 3C.

The behaviors of these two DNAzymes have been rationalized by a unified model (Figure 3D), in which a maximum catalytic activity is reached at optimal mechanical stability of each DNAzyme. At the low force regime below the maximum activity, the higher the external force, the weaker the DNAzyme structure and therefore, the higher the catalytic activity. On the other hand, at the high force regime above the maximum activity, further increase in the external force may damage the GQ structure, driving hemin out of the GQ framework, reducing the catalytic activity. This unprecedented force modulation on catalytic activities led us to propose a new type of catalyst, mechanozyme, in which the structure of the catalyst is made of a mechanophore (such as GQ) [27, 28, 29, 30] responsive to external forces.

The mechanical modulation allows us to obtain mechanozymes with superior catalytic activities. For the AR→RF fluorogenic reaction described in Figure 1A, the GQ‐11oxoG‐hemin at an external force of 1 pN shows substantially greater catalytic efficiency (7.31 s^−1^µm ^−1^ (Figure 2F)) than known nanozymes (0.04–1.60 s^−1^µm ^−1^, Table S7) [39, 40, 41] or DNAzymes (0.12–2.32 s^−1^µm ^−1^, Table S7) [14, 42]. In fact, it is located at the upper end of natural peroxidases such as HRP [43] (0.014–8 s^−1^µm ^−1^, Table S7). The pivotal component for superior activity is the weakened GQ structure of the GQ‐11oxoG‐hemin under mechanical force, which demonstrates the power of the marginal stability to govern mechanozyme activities.

### 2.4 Sonomechanical Modulation of a Large Population of Mechanozymes

The high‐resolution single‐molecule mechanical experiments confirmed the marginal stability principle in artificial enzymes, leading to the mechanozyme concept. However, application of force on mechanozymes using MT‐HILO or other single‐molecule techniques is not practical due to their low throughput. Ultrasonication has been used to mechanically alter biomolecular structures [44, 45, 46] due to the shear forces associated with cavitations of nanobubbles generated by ultrasonication [47]. As ultrasonication can be applied to an entire solution, it is ideal to exert sonomechanical force on a massive set of molecules simultaneously [45, 48, 49]. Therefore, we employ ultrasonication to achieve mechanical modulations on mechanozyme activities at the bulk level.

**FIGURE 4:**
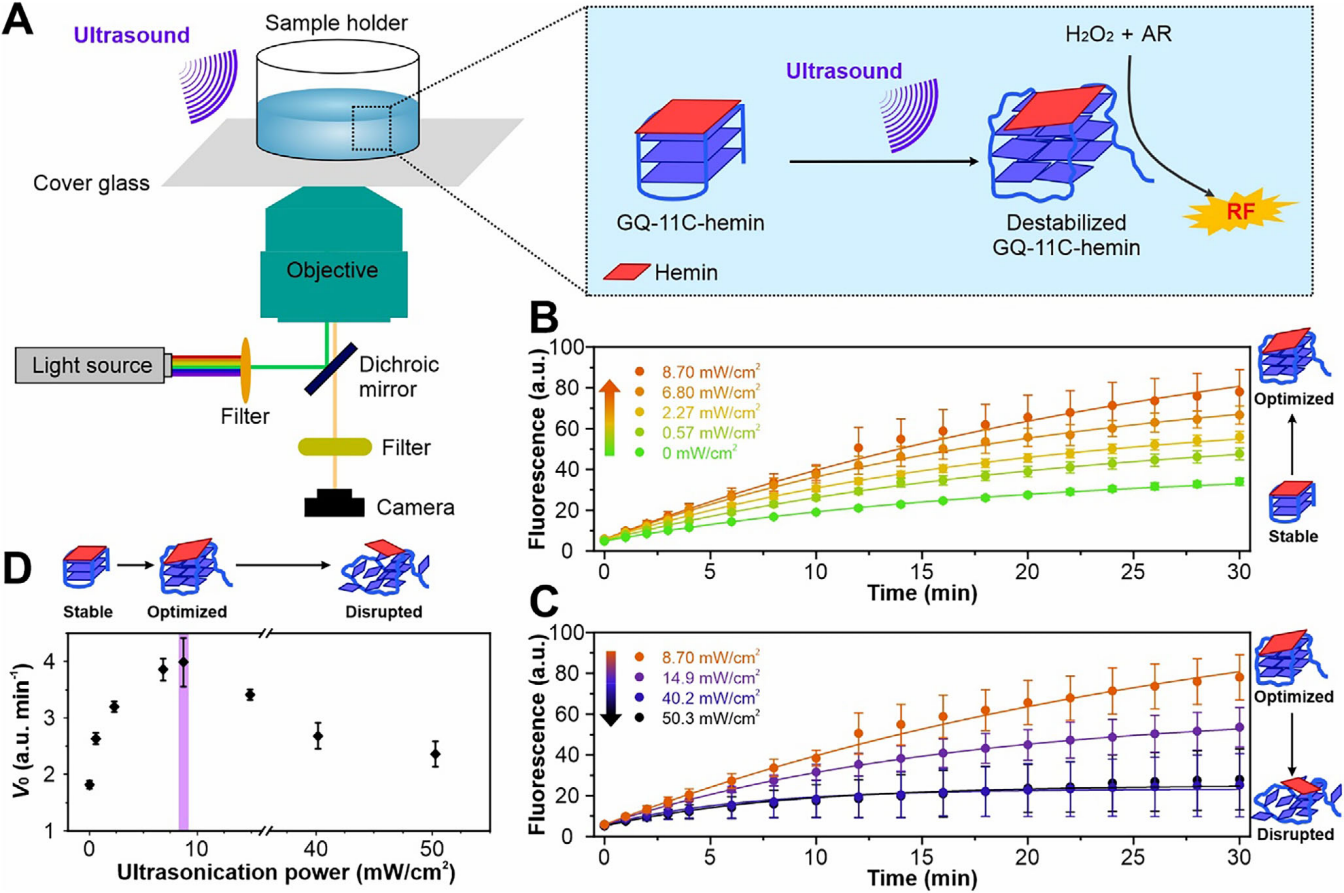
Sono‐mechanical force modulates activities of an ensemble set of GQ‐11C‐hemin mechanozymes. (A). Setup of experiments at the bulk level (details see Section S8 and Figure S17). (B, C). The fluorescence intensity versus time of GQ‐11C‐hemin under different ultrasonication powers (frequency 10 kHz). (D). *V*_0_ of GQ‐11C‐hemin under different ultrasonication powers (details see Sections S4 and S8). The data was fitted by a single‐exponential equation to guide the eyes. Error bars depict the standard deviations from three independent experiments. The reactions were performed at 25°C with 0.5 mm h_2_ o_2_ and 10 µm AR in a sucrose buffer (40% (w/v) sucrose, 100 mm KCl, 10 mm Tris at pH 7.4).

We used the same reaction system (AR to RF) shown in Figure 1A to monitor the activities of GQ‐11C‐hemin. This DNAzyme was chosen based on its medium thermostability (Figure 1C). While WT Tel‐4G may be too stable to respond to the ultrasonication, other mutants, such as GQ‐11A, could be too unstable to bind hemin under ultrasonication force. The reaction solution was then ultrasonicated (Figure 4A) with a cylindrical microtip transducer probe at different powers (see Section S8 and Figure S17 for details).

If the sono‐mechanical force [48] generated by ultrasonication would reduce the stability of GQ‐11C‐hemin, then we expect the activity of the DNAzyme should increase with ultrasonication power. When the power exceeds a threshold value, we anticipate that the GQ structure is disrupted such that hemin is no longer bound, causing a decrease in activity. Figure 4B,C shows temporal fluorescence intensity traces of the RF product at different ultrasound powers. To assess activity, we used initial velocity (*V*_0_) to compare GQ‐hemin activities (see Section S4 under the subheading “The initial velocity (*V*_0_) calculations” for details). The experimental results aligned with the prediction: *V*_0_ increased with ultrasound power from 0 to 8.70 mW/cm^2^ but decreased at higher power levels (Figure 4D; Figures S18–S20 and Table S6). Furthermore, the reduction in catalytic activity observed at higher ultrasonic power (8.70–50.3 mW/cm^2^) was effectively restored to its original level after the mechanozyme subjected to 50.3 mW/cm^2^ ultrasonic power was tested without ultrasound. (see Section S8 and Figures S21 and S22 for details).

To confirm whether ultrasonication indeed reduces GQ stabilities, we performed a fluorescence‐based melting temperature (*T*_m_) measurement of the GQ‐11C with and without 460 mW/cm^2^ ultrasonication (details see Section S9). We found that the *T*_m_ under ultrasonication (18.5 ± 0.4°C) was significantly lower than that without ultrasonication (26.0 ± 0.8°C, which is consistent with the *T*_m_ of 28.4 ± 0.3°C obtained from the 295 nm UV melting experiment, see Figures S24 and S25), indicating that ultrasonication indeed weakened the structural stability of the GQ. In addition, control experiments confirmed that radicals other than H_2_O_2_ produced by the ultrasonication had an insignificant contribution to enhance catalytic activities (see Figure S23).

Using Michaelis–Menton kinetics (see Section S10), we estimated the activity efficiency of the GQ‐11C‐hemin mechanozyme to catalyze the AR→RF conversion under ultrasonication. We found that under optimized sono‐mechanical stress of 8.70 mW/cm^2^ (Figure 4B,C), this mechanozyme had a catalytic efficiency of ∼9.73 s^−1^µm ^−1^. This value surpasses all known natural or synthetic peroxidases to the best of our knowledge (see Tables S7 and S8 for comparison of activities), demonstrating unprecedented efficiency of mechanical modulations to enhance mechanozyme activities.

## 3 Conclusion

We have used DNAzymes to firmly establish that the marginal stability principle exists in artificial enzymes. Compared to natural enzymes, DNAzymes have much simpler structures. This suggests that marginal stability is a universal phenomenon that governs enzymatic activities across different biomacromolecules. As the stabilities of biomacromolecules can be altered by an external force along a specific direction, application of the mechanical force on biomolecules can modulate their enzymatic and even other activities responsive to the magnitude and/or directionality of applied forces. Using ultrasound‐based mechanical modulation, we have successfully demonstrated that a massive set of GQ‐hemin mechanozymes shows catalytic efficiency superior to natural or synthetic peroxidase. We anticipate that the proposed mechanozymes serve as a general concept in which their catalytic activities can be modulated by mechanical forces. This property can be readily extended to any biomolecules or mechanophores with force‐responsive activities beyond catalytic actions.

## Funding

National Institutes of Health (NIH) (R01 CA252827), National Science Foundation (NSF) (Che 2247709), Kent State University's Farris Family Foundation, and Graduate Student Senate (GSS) Research Award.

## Conflicts of Interest

The authors declare no conflicts of interest.

## Supporting information




**Supporting File**: advs75254‐sup‐0001‐SuppMat.docx.

## Data Availability

The data that support the findings of this study are available in the supplementary material of this article.
