# Supplementary material for: Mechanozyme: An Artificial Enzyme With a Mechanophore Framework
Source: Adv Sci (Weinh). 2026 Apr 20;13(39):e75254. doi: 10.1002/advs.75254 (PMC13334946; doi:10.1002/advs.75254)
Supplement: Supplementary file 1 — Supporting File: advs75254‐sup‐0001‐SuppMat.docx. [file ADVS-13-e75254-s001.docx]

**Supporting Information**

**Mechanozyme: an artificial enzyme with a mechanophore framework**

Jiahao Ji,^1^ Pravin Pokhrel,^1^ Sajan Shakya,^1^ Bishal Pokhrel,^1^ Grinsun Sharma,^1^ Pratiksha Chaudhary,^1^ Hao Shen,^1^ and Hanbin Mao^1,2,3,^*

^1^Department of Chemistry & Biochemistry, Kent State University, Kent, OH 44242, USA

^2^Advanced Materials and Liquid Crystals Institute, Kent State University, OH 44242, USA

^3^School of Biomedical Sciences, Kent State University, OH 44242, USA

^*^ Corresponding author: Hanbin Mao (hmao@kent.edu)

Table of contents

Contents

[S1. Materials and methods 3](#_Toc178153057)

[S2. Circular Dichroism (CD) spectra](#_Toc178153057) 9

[S3. UV melting experiments 10](#_Toc178153057)

[S4. Fluorescence bulk experiments and *V*_0_ calculations 11](#_Toc178153057)

[S5. Synthesis of DNA samples 16](#_Toc178153058)

[S6. Single-molecule fluorescent MT-HILO microscopy 17](#_Toc178153058)

[S7. Single-molecule unfolding using optical tweezers 20](#_Toc178153058)

[S8. Fluorescence bulk experiments under ultrasonication 34](#_Toc178153058)

[S9. Sono-melting experiments 45](#_Toc178153059)

[S10. Summary of enzyme activities and catalytic properties 48](#_Toc178153059)

[S11. References 52](#_Toc178153059)

## S1. Materials and Methods

**Materials**

All chemicals, unless specified, were purchased from Sigma Aldrich ([www.sigmaaldrich.com](http://www.sigmaaldrich.com)), Thermo Fisher (www.thermofisher.com), or VWR ([www.vwr.com](http://www.vwr.com)). DNA oligos without oxoG modification were purchased from Integrated DNA Technologies (www.idtdna.com) and purified by PAGE (sequence details see Table S1). The oxoG modified DNA oligos were bought from Eurogentec (www.eurogentec.com) or Midland Certified Reagent Co. (Midland, TX, USA) and purified by HPLC. DNA enzymes and BSA were obtained from New England Biolabs (www.neb.com). The anti-digoxigenin coated polystyrene beads (diameter ~2.3 µm) and streptavidin coated polystyrene beads (diameter ~1.8 µm) were bought from Spherotech (Lake Forest, IL, USA). The Dynabeads™ M-270 streptavidin-coated superparamagnetic beads (diameter ~2.8 µm) were acquired from Life Technologies (Carlsbad, CA, USA). Nitrocellulose was bought by Cytiva Life Sciences (Marlborough, MA, USA). The DI water used to prepare solutions and buffers was obtained from CANSHI^®^ Ultrapure water system (Zhejiang Canshi, China).

**Methods**

**Initial velocity (*V*_0_) measurements for DNAzymes**

Catalytic activities of different GQ-hemin DNAzymes were measured in an inverted fluorescent microscope (Nikon TE2000-U) and the initial velocity (*V*_0_) was calculated via fluorescence intensity versus time curves.^[^[^1^](#_ENREF_1)^]^ First, each GQ mutant sequence (25 µM) was incubated with hemin (25 µM) at 1:1 ratio with sucrose buffer (40% (w/v) sucrose, 100 mM KCl, and 10 mM Tris, pH 7.4), which was put in 4 °C overnight. Then, 0.5 µL GQ-hemin (25 µM), 5 µL Amplex Red (AR) (100 µM), 2.5 µL H_2_O_2_ (10 mM) and sucrose buffer were quickly mixed, forming 50 µL reaction buffer (0.25 µM GQ-hemin, 0.5 mM H_2_O_2_ and 10 µM AR). The reaction buffer was added to a microfluidic chamber and fluorescent signals from resorufin (excitation ~571 nm, emission maximum, ~583 nm) were detected under the fluorescent microscope. To reduce errors, the time interval between reactant preparation and fluorescence signal detection was kept at 1 min. The fluorescent signal versus time curves were fitted by exponential equation and the slopes of the fitted exponential curves at initial time represented the initial velocity (*V*_0_) for each GQ-hemin. These slopes had similar values with those directly measured from the first 4 minutes of each temporal fluorescent intensity trace.

**UV melting**

UV melting experiments were conducted using a Varian Cary 300 spectrophotometer (Agilent Technologies, USA) with a quartz cuvette having a 1 cm optical path length. Prior to measuring the UV melting temperature (*T*_m_), 5 µM DNA samples were prepared in 500 µL sucrose buffer (40% (w/v) sucrose, 100 mM KCl, and 10 mM Tris, pH 7.4). The samples were heated to 95°C for 10 minutes, then rapidly cooled on ice. During the measurement, absorbance was recorded at 295 nm following established protocols.^[^[^2-3^](#_ENREF_2)^]^ The *T*_m_ values of various GQ mutants are presented below (Supporting Information Section S3).

**Single-molecule fluorescent experiments by MT-HILO microscopy**

The single-molecule fluorescent MT-HILO (magnetic tweezers coupled with highly inclined and laminated optical sheet) (Figure S6) was performed on an Olympus IX83 inverted microscope, which has been described in literature.^[^[^4-5^](#_ENREF_4)^]^ In brief, two cubic permanent magnets (6 mm NdFeB magnets coated with Nickel, Supermagnete, Germany), separated by a 0.5 mm gap, were mounted in a custom-designed aluminum holder to generate magnetic forces on superparamagnetic beads (2.8 µm diameter; Dynabeads™ M270 Streptavidin, Thermo Fisher Scientific, Waltham, MA, USA) (Figure 2A). A 532 nm continuous laser (MGL-III-532, 300 mW; Dragon Laser, Changchun, Jilin, China) was used as the excitation source. The laser beam was directed and modulated via a Highly Inclined and Laminated Optical sheet (HILO) mirror, enabling excitations in either epi-fluorescence or HILO excitation.^[^[^6-7^](#_ENREF_6)^]^ Calibration of the tensile force applied to the sample was performed as previously described and shown in Section S6.

In single-molecule fluorescence experiment of GQ-hemin catalysis, we first covered 50 µL 0.1% nitrocellulose on the surface of a piranha cleaned glass coverslip (VWR, 24 mm width × 50 mm length × 0.13–0.16 mm thickness). After the coverslip was dried at room temperature, we heated the coverslip at 120 °C for 5 minutes, allowed it to cool to room temperature, and then assembled it into a one-channel microfluidic chamber using a piece of glass coverslip (VWR, 24 mm width × 60 mm length × 1 mm thickness), double-sided tape, and epoxy glue (Figure S7). Then, 0.02 µg/µL anti-digoxigenin in 50 µL buffer solution (50 mM potassium phosphate buffer, pH 7.4) was injected into the chamber and incubated for around three hours, where anti-digoxigenin was fixed to the nitrocellulose-coated inner surface of coverslip. The nitrocellulose-coated surface of the coverslip was later passivated by 50 µL 5 mg/mL BSA-containing buffer (50 mM potassium phosphate buffer, pH 7.4) for 16 hrs. DNA (1 µL 0.01 nM DNA) and hemin mixed samples were incubated with extra streptavidin-coated superparamagnetic beads (20 µL at 6–7 ×10^8^ beads/mL) for ~30 minutes (final hemin concentration 25 µM) in a buffer of 40% (w/v) sucrose, 100 mM KCl, and 10 mM Tris (pH 7.4). Prior to injection into the microfluidic chamber, the sample and bead mixture were washed 2–3 times with the same sucrose buffer mentioned above to remove excess free hemin molecules. Following injection, the sample was incubated for an additional 30 minutes.

Before measuring catalytic activities of DNAzymes, the pair of magnets were rotated to verify the circular rotational patterns of the superparamagnetic beads, thereby confirming the presence of single DNA tethers between the coverslip and the magnetic bead, as described previously.^[^[^4^](#_ENREF_4)^,^ [^8^](#_ENREF_8)^]^ The distance of the magnets to the microchamber surface was adjusted as needed to apply specific mechanical forces to the samples (Figure S8). Calibration of the distance versus mechanical forces resulted in an equation, $Y=A*e^{\left( -\frac{x}{t} \right)}+Y_{0}$, where *Y* represents the applied force, and *x* denotes the distance between the cover glass and the magnets. Due to the MT-HILO design, non-catalytic events occurring on the chamber surface were completely out of focus (Figures S6-S7). To minimize background fluorescence, residual signals from each superparamagnetic bead were photobleached for at least 30 minutes using the same 532 nm laser for resorufin excitation. During movie acquisition of fluorescence signals, the exposure time was set to 50 ms per frame. The reaction buffer (3 mM H_2_O_2_, 50–1500 nM AR, 40% (w/v) sucrose, 100 mM KCl, and 10 mM Tris (pH 7.4) was introduced at a flow rate of 1 µL/min at 25 °C. Movies were processed using ThunderSTORM.^[^[^9-10^](#_ENREF_9)^]^ Data analysis procedures, including reaction rate calculations, followed established protocols described in the literatures.^[^[^11-12^](#_ENREF_11)^]^

**Single-molecule mechanical unfolding in optical tweezers**

A thorough description of the home-made optical tweezers (OT) instrument has been reported in previous literature.^[^[^13^](#_ENREF_13)^]^ Briefly, a 1064 nm diode pumped solid-state (DPSS) laser (4 W, continuous-wave mode, BL-106C, Spectra-physics) was split to generate two laser beams, forming two laser traps. The positions of the two traps were independently monitored using position-sensitive photodetectors (PSD; DL100, Pacific Silicon Sensor), while one of traps was actively controlled via a steerable mirror (Nano-MTA, Mad City Labs, Madison, WI).

In optical tweezers-related experiments (Figure S9), DNA sample was prepared based on the reported method (Supporting Information Section S5) and a four-channel microfluidic chamber (Figure S10) was employed to conduct single-molecule mechanical folding/unfolding studies. A patterned sheet of paraffin film (Parafilm M, Bemis Laboratory Film, Bemis North America, WI, USA; thickness approximately 130 µm) was prepared using a laser cutter (VersaLaser®, Universal Systems, Inc., UT, USA). This paraffin film was sandwiched between two cover glasses (VWR; 24 mm × 60 mm; thickness 0.13–0.16 mm), with eight laser-cut holes on one of the cover glasses to achieve buffer exchange. Following assembly, the chamber was heated at 95 °C to melt the paraffin film, thereby creating a tight seal between two cover glasses. Two capillary tubes (King Precision Glass, Inc., PO MC 7701; inner diameter 0.025 ± 0.010 mm) were inserted between microchannels to establish interconnections within the chamber.

During single-molecule mechanical folding/unfolding experiments, DNA samples (Figure S5) were initially mixed with anti-digoxigenin (dig)-coated polystyrene beads and incubated at 25 °C for 30 minutes to facilitate DNA attachment to the beads. The bead–DNA complexes were then introduced into the top channel, which were transported into the middle channels via a capillary tube (Figures S9-S10). Streptavidin-coated beads were subsequently injected into the bottom channel and later released to middle channels through the other capillary tube (Figure S10). Once a streptavidin-coated bead and a DNA–anti-digoxigenin-coated bead were individually trapped by two laser foci, these two beads were brought into close proximity to enable tethering of a single DNA molecule between them (Figures S9-S10). This configuration was then subjected to force-ramping protocols to perform mechanical unfolding/folding measurements as described.

During the force-ramping process, the tethered DNA molecule was stretched/relaxed by moving one bead (normally anti-digoxigenin (dig)-coated polystyrene beads) away from or towards another. The DNA sample was stretched/relaxed in the buffer-only middle microchannel and then moved to the other middle microchannel containing hemin molecules. During this process, force versus extension (F-X) traces were recorded and then analyzed by Labview software (National Instruments, TX). After one whole stretching-relaxing process, the optical traps were held stationary for a defined incubation period, typically 30 seconds, to allow the formation of DNA secondary structures. The flow speed of buffers was maintained at 0.5 µL/min during experiments performed at 25 °C. The loading rate during experiments was kept at ~5.5 pN/s (in the 10–30 pN force range).

**Table S1.** List of DNA oligonucleotides sequences used in this work.

| **Name** | **Sequences (5’- to -3’)** |
| --- | --- |
| Tel-4G mutant G-oxoG (GQ-11oxoG sequence) | CGGTACGGTGTGAAATACGCGAGAAAGGAATTAGGGTTAG/oxoG/GTTAGGGTTAGGGTTATCCGCGCGGATTGCCTGAGCACGTC |
| WT Tel-4G GQ  (WT Tel-4G sequence) | CGGTACGGTGTGAAATACCGCACAGATGCGTTAGGGTTAGGGTTAGGGTTAGGGTTAGCCAGCAAGACGTAGCCCAGCGCGTC |
| Primer 1 | TTCCTTTCTCGCGTATTTCACACCGT |
| Primer 2 | CAGGGACGTGCTCAGGCAATCCGCGCGGA |
| Primer 3 | CGCATCTGTGCGGTATTTCACACCGT |
| Primer 4 | CAGGGACGCGCTGGGCTACGTCTTGCTGGC |
| GQ-11oxoG for CD/UV/Bulk | TTAGGGTTAG/oxoG/GTTAGGGTTAGGGTTA |
| WT Tel-4G GQ for CD/UV/Bulk | TTAGGGTTAGGGTTAGGGTTAGGGTTA |
| Tel-4G mutant 11A  (GQ-11A) | TTAGGGTTAG**A**GTTAGGGTTAGGGTTA |
| Tel-4G mutant 11C  (GQ-11C) | TTAGGGTTAG**C**GTTAGGGTTAGGGTTA |
| Tel-4G mutant 11T  (GQ-11T) | TTAGGGTTAG**T**GTTAGGGTTAGGGTTA |
| Random ssDNA for  internal standard | CTGTGAGGTAGTAGCAACATATCAACCTCAAGGAGCTTCAGT CTAGCG |

## S2. Circular Dichroism (CD) spectra

Circular Dichroism (CD) spectra were acquired using a Jasco J-810 spectropolarimeter (Easton, MD). DNA samples (10 µM) were prepared in 200 µL of various buffers. To ensure proper folding, samples were denatured at 95 °C for 10 minutes and rapidly cooled on ice. CD measurements were performed in a 1 mm path length quartz cuvette, with each spectrum averaged from three consecutive scans collected at 1 nm/s over a 200–320 nm wavelength range. Background spectra of the corresponding buffers were subtracted to correct for baseline interference.

**
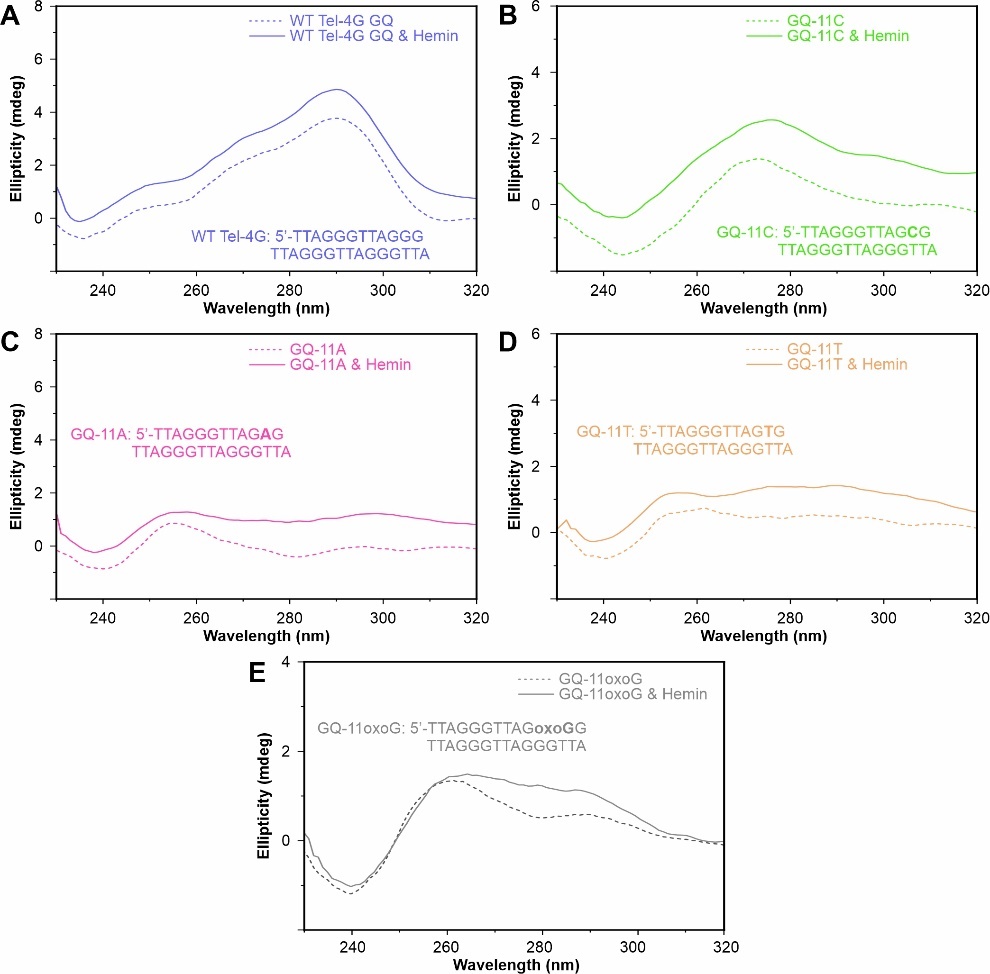
**

**Figure S1.** CD spectra of different GQ sequences (see Table S1 for details) without and with hemin. (Buffer: 40% (w/v) sucrose, 100 mM KCl, 10 mM Tris, pH 7.4; with hemin: 25 µM hemin, 40% (w/v) sucrose, 100 mM KCl, 10 mM Tris, pH 7.4).

## S3. UV melting experiments


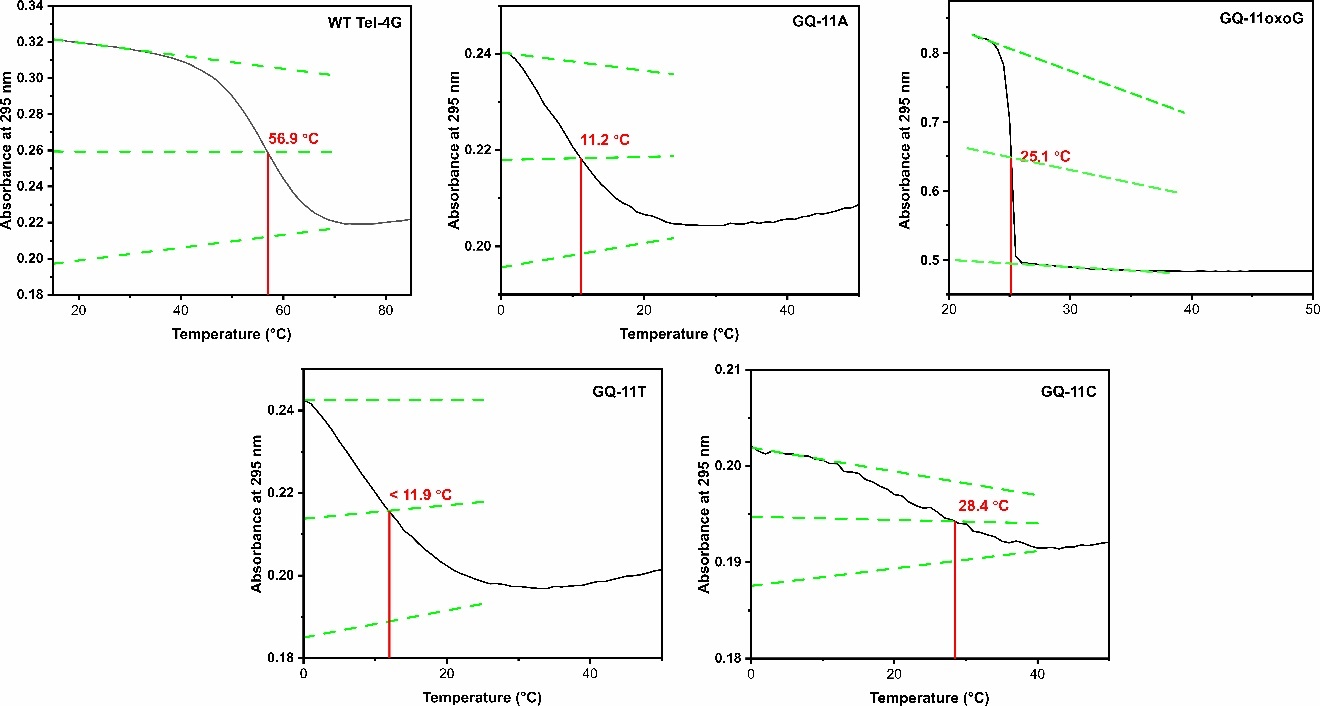


**Figure S2.** Typical UV melting curves of 5 µM different GQ sequences (see Table S1 for details) in sucrose buffer (40% (w/v) sucrose, 100 mM KCl, 10 mM Tris, pH 7.4) at 295 nm with a temperature ramping at 0.5 ℃/minute.

## S4. Fluorescence bulk experiments and *V*_0_ calculations

**Sample preparation**

To prepare GQ-hemin complexes, DNA molecules of various G-quadruplexes (WT Tel-4G or other mutants) were mixed with hemin at a 1:1 molar ratio ([DNA]: 25 µM; [hemin]: 25 µM) in sucrose buffer. The mixture was heated to 95 °C for 10 minutes, rapidly cooled on ice, and stored at 4 °C. GQ formation was verified by Circular Dichroism (CD) spectroscopy (see Section S2).

For fluorescence bulk experiments, 0.5 µL GQ-hemin (25 µM), 5 µL AR (100 µM), 2.5 µL H₂O₂ (10 mM), and sucrose buffer (40% (w/v) sucrose, 100 mM KCl, and 10 mM Tris, pH 7.4) were rapidly mixed to prepare a 50 µL reaction sample (final concentrations: 0.25 µM GQ-Hemin, 10 µM AR, 0.5 mM H₂O₂). To ensure consistency, the interval between preparation of reaction mixture and initiating data collection (t = 0 min) was standardized to approximately 1 minute.

**Fluorescence bulk experiment setup**

The reaction buffer was added to a modified glass container (sample holder: 5 mm diameter & 10 mm height), and the fluorescence signals from the reaction buffer were measured on an inverted fluorescence microscope (Nikon TE2000-U) (see Figure S3).

**
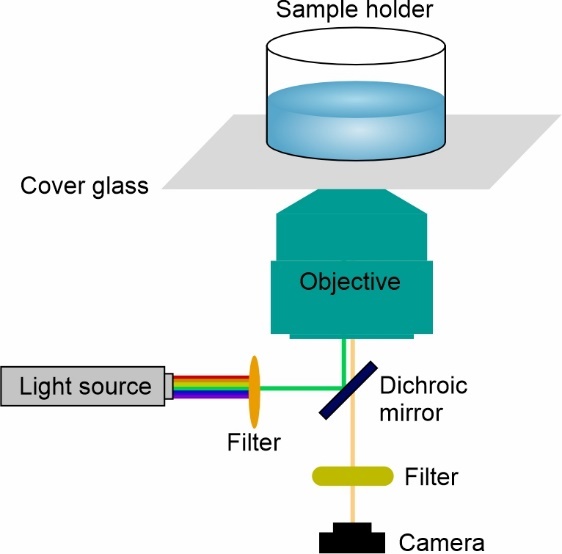
**

**Figure S3.** Setup of fluorescent bulk experiments for the AR🡪RF reaction catalyzed by different DNAzymes (Figure 1A).

**The initial velocity (*V*_0_) calculations**

Fluorescence signal versus time curves from Figure 1B were fitted to either an exponential equation ($F=F_{0}+A*e^{R_{0}*t}$, Figure S4 left) or a linear equation ($F=a+b*t$ , Figure S4 right) to determine the initial reaction velocity (*V*_0_).

After fitting with a single exponential equation, the initial rate velocity (*V*_0_) was determined from the slope of the fitted curves at t = 0 min.

After fitting with a linear equation over the first four minutes of the reaction, the slope of the fitted curve depicts the initial rate (*V*_0_).

The fluorescence intensity (arbitrary unit or a.u.) was plotted against time (t, in minutes). The values of initial velocity (*V*_0_) are presented in Table S2, with both exponential and linear fitting results exhibiting consistent trends.

**
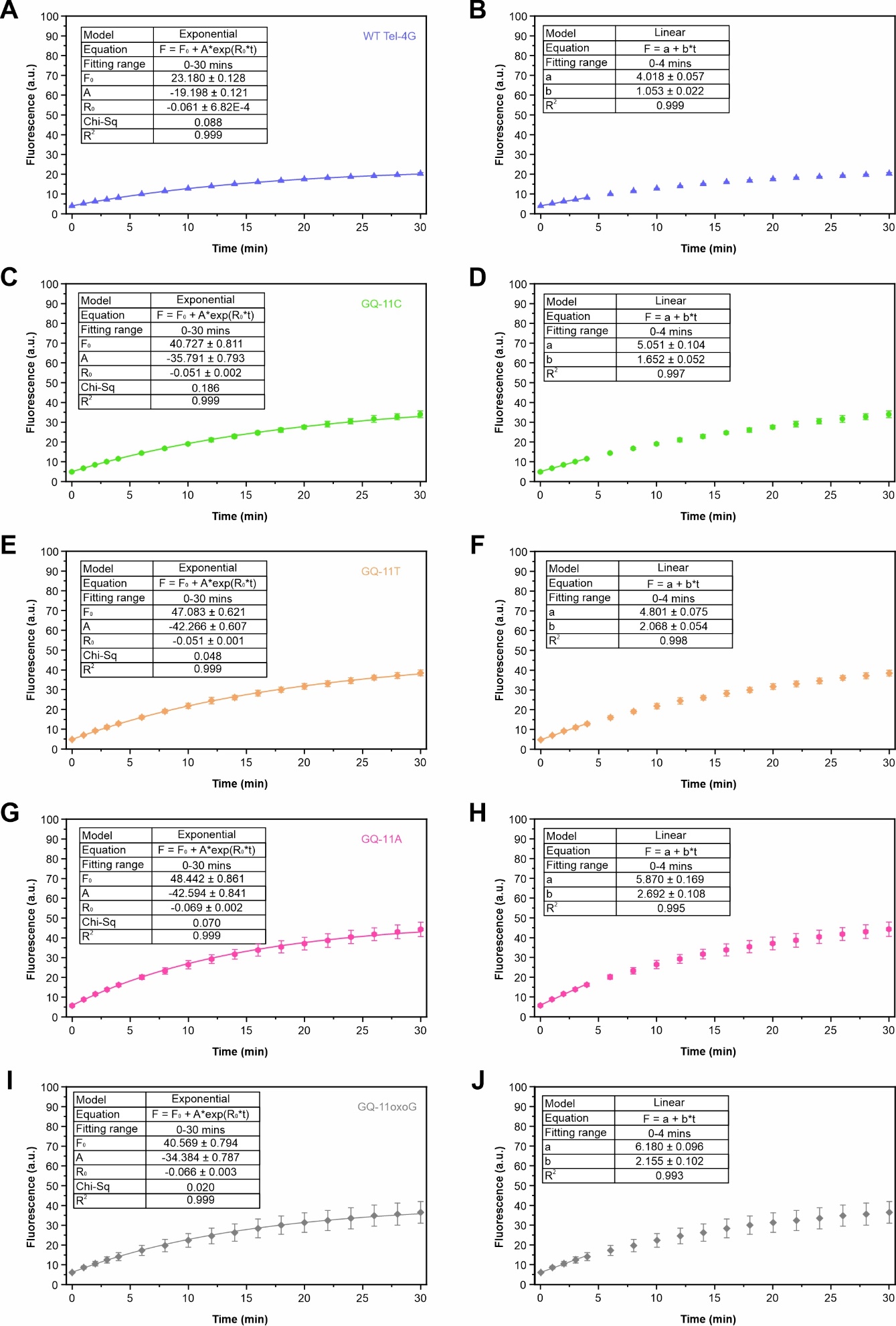
**

**Figure S4.** Fluorescence intensity vs time curves of the AR🡪RF reaction (Figure 1A) catalyzed by different DNAzymes described in Figure 1. The WT Tel-4G GQ-hemin curves were fitted with a single exponential (A) or linear equation (B). Curves of GQ-11C-hemin (C, D), GQ-11T-hemin (E, F), GQ-11A-hemin (G, H) and GQ-11oxoG-hemin (I, J) were fitted with single exponential (left panels) or linear equations (right panels).

**Table S2.** Initial velocity (*V*_0_, (a.u)/min) of different GQ-hemin complexes (see Figures 1B and S4 for raw data).

| **Sample** | ***V*_0_**  **(Exponential fitting)** | ***V*_0_**  **(Linear fitting)** |
| --- | --- | --- |
| WT Tel-4G GQ-hemin | 1.17 ± 0.02 | 1.05 ± 0.02 |
| GQ-11C-hemin | 1.82 ± 0.07 | 1.65 ± 0.05 |
| GQ-11T-hemin | 2.16 ± 0.06 | 2.07 ± 0.05 |
| GQ-11oxoG-hemin | 2.28 ± 0.10 | 2.16 ± 0.10 |
| GQ-11A-hemin | 2.92 ± 0.11 | 2.69 ± 0.11 |

## S5. Synthesis of DNA samples

To prepare DNA samples for MT-HILO and optical tweezers (OT) experiments, we first phosphorylated the WT Tel-4G GQ or GQ-11oxoG sequence and oligo a (Step 1; see Figure S5 and Table S1 for sequences). The phosphorylated GQ sequence, phosphorylated oligo a, and oligo b were mixed at a 1:1:1 ratio, heated to 95°C for 5 minutes, and gradually annealed from 95 °C to 20 °C at a rate of 0.5 °C per minute (Step 2). The annealed sample was then ligated with 1558 bp and 2391 bp dsDNA handles at a 1:1:1 ratio in a one-pot reaction using T4 DNA ligase (New England Biolabs, US) at 16 °C for 18 hours (Step 3). The ligase was deactivated by heating to 65°C for 20 minutes. The ligated DNA samples were stored at –20 °C until use.

The preparations of the 1558 bp and 2391 bp dsDNA handles were carried out following methods described in previous literatures.^[^[^14-15^](#_ENREF_14)^]^


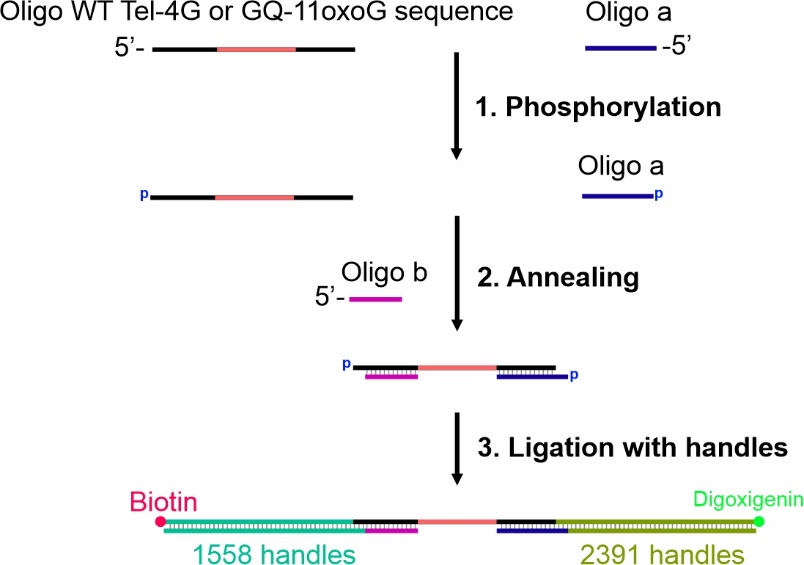


**Figure S5.** Syntheses of DNA samples for the Optical Tweezers (OT) and MT-HILO experiments. For GQ-11oxoG sample preparation, oligo a is the primer 2 and oligo b is the primer 1 in Table S1. For WT Tel-4G GQ sample preparation, oligo a is the primer 4 and oligo b is the primer 3 in Table S1.

## S6. Single-molecule fluorescent MT-HILO microscopy

**
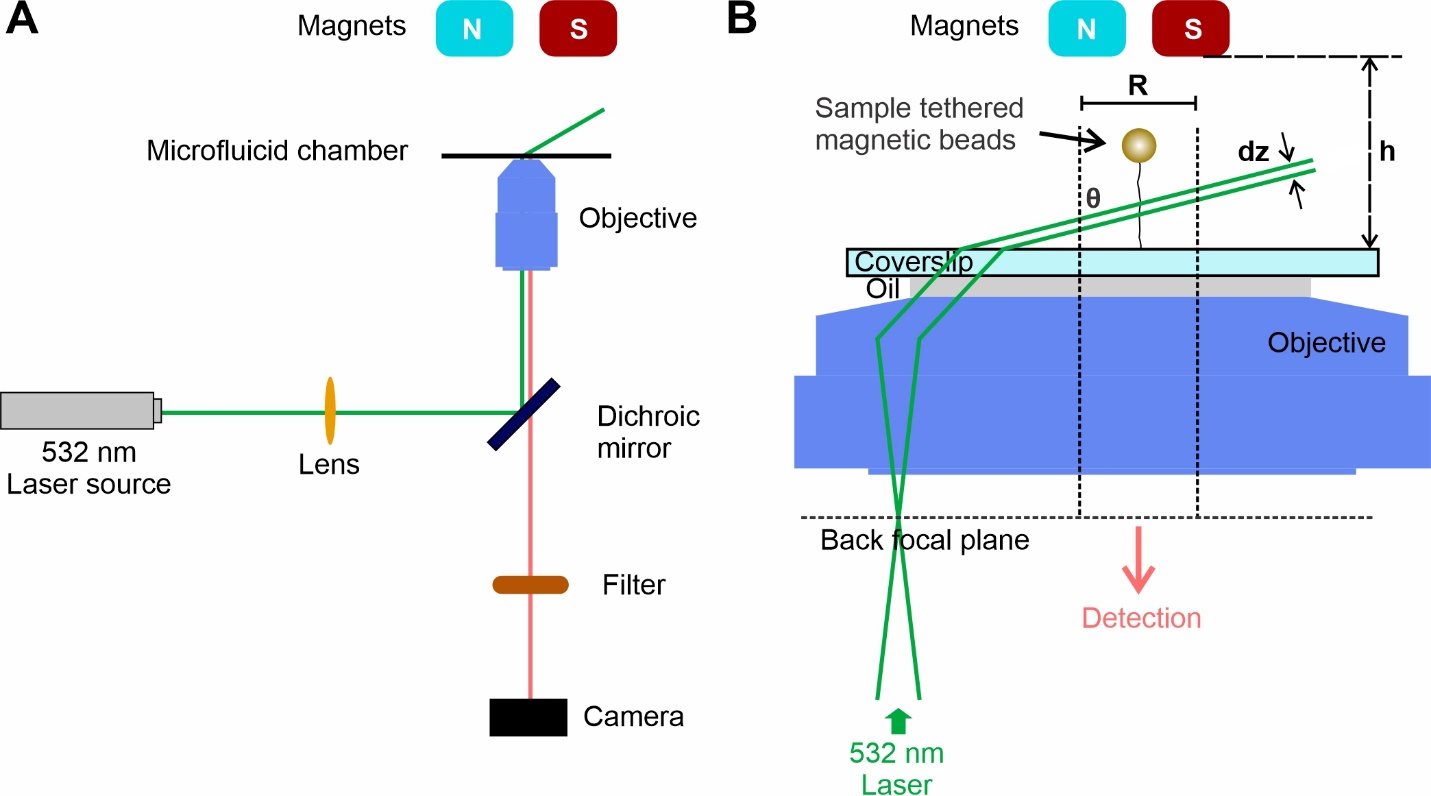
**

**Figure S6.** Setup of the MT-HILO. (A) Layout of the MT-HILO microscope. (B) HILO excitation of a single GQ-hemin complex. Here, R denotes the diameter of the illuminated region (10 μm), and θ represents the incidence angle at the coverslip (~70°). The laser-illuminated thickness (dz), is calculated as dz = R/tanθ, yielding approximately 3.6 μm. The h indicates the distance between the chamber’s coverglass and the magnets. The calibration curve relating the applied force to the distance is presented in Figure S8.

**
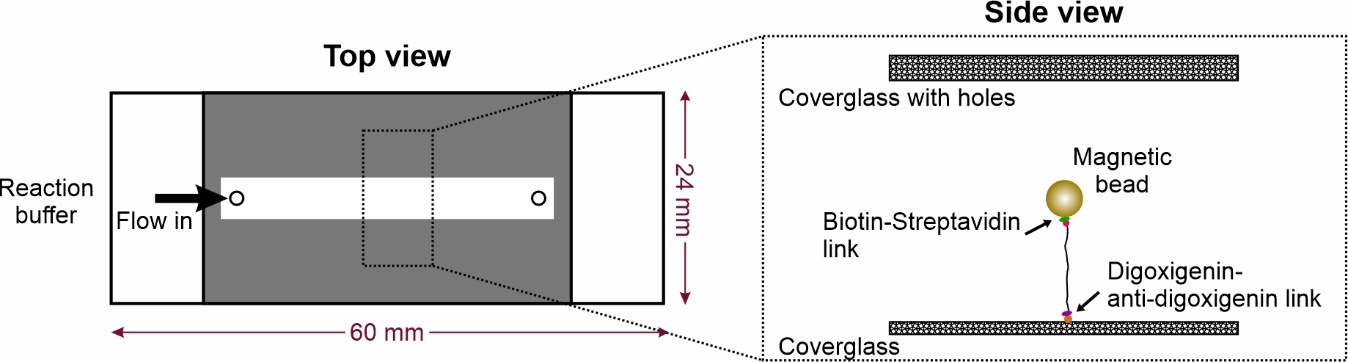
**

**Figure S7.** Microfluidic chamber for the MT-HILO experiments. Thickness of each chamber is ~1.25 mm.


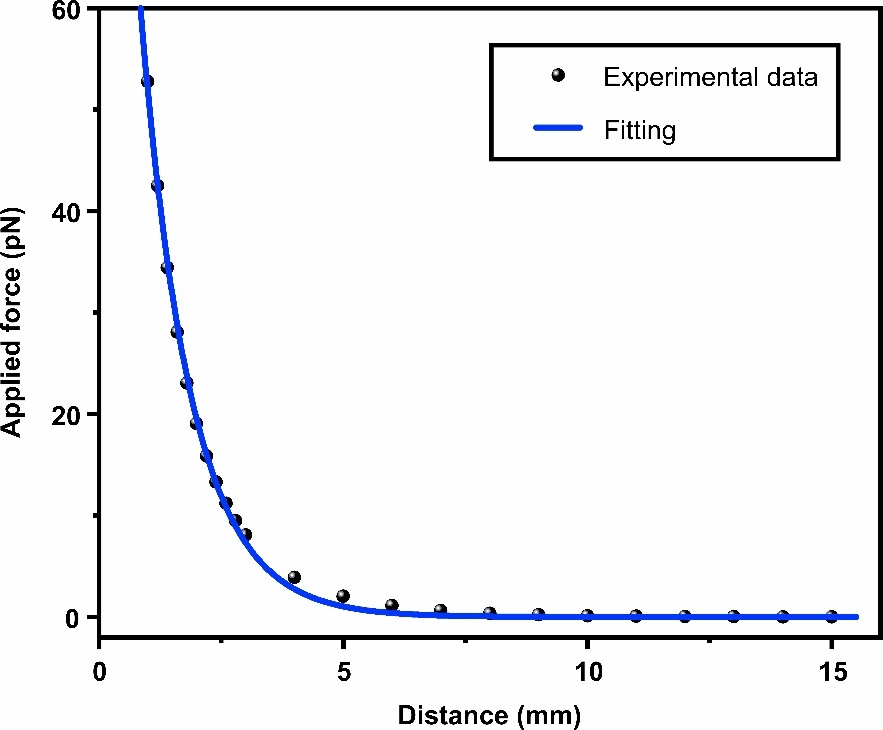


**Figure S8.** The applied force on the superparamagnetic bead versus the distance between the pair of magnets and the chamber surface used in the MT-HILO experiments. The exponential equation ($Y=A*e^{\left( -\frac{x}{t} \right)}+Y_{0}$) was used to fit the data, here *x* represents the distance (mm), *Y* denotes the applied force (pN), *A* = 137.686 ± 2.800, *t* = 1.023 ± 0.015, and *Y*_0_ = 0).

## S7. Single-molecule unfolding using optical tweezers

Optical tweezers are a well-developed single-molecule force spectroscopy technique that uses a tightly focused laser beam to trap and manipulate microscopic dielectric particles, such as polystyrene beads (Figure S9). In our experiments, the single-molecule nature of optical tweezers enables mechanical unfolding and refolding measurements with sub-nanometer spatial resolution^[^[^16^](#_ENREF_16)^]^ and millisecond temporal resolution^[^[^17-18^](#_ENREF_17)^]^. Over the past decades, this technique has allowed precise quantification of the mechanical stability of diverse biomolecules,^[^[^19^](#_ENREF_19)^]^ including G-quadruplexes,^[^[^17^](#_ENREF_17)^]^ DNA hairpins,^[^[^20^](#_ENREF_20)^]^ and proteins,^[^[^21^](#_ENREF_21)^]^ with piconewton-level force sensitivity.

**Optical tweezers setup**

**
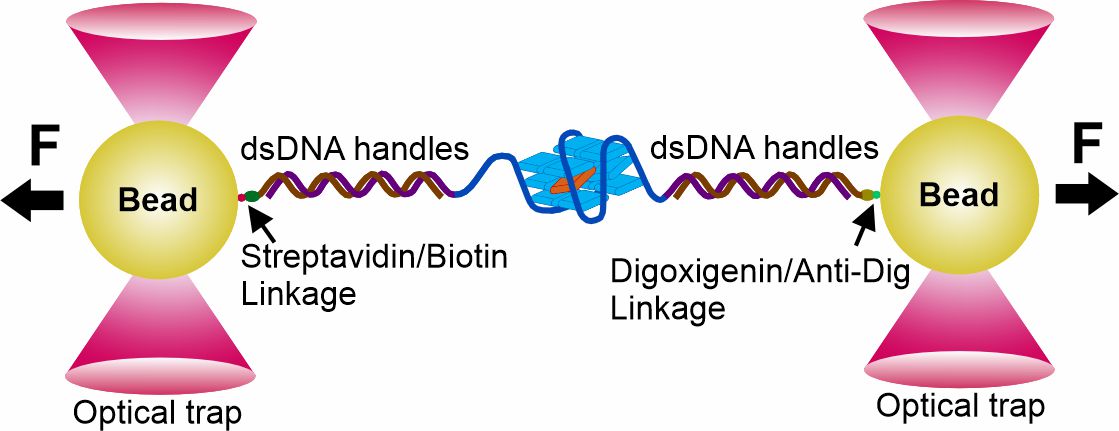
**

**Figure S9.** Setup of mechanical unfolding of WT Tel-4G/GQ-11oxoG using optical tweezers (OT).

**
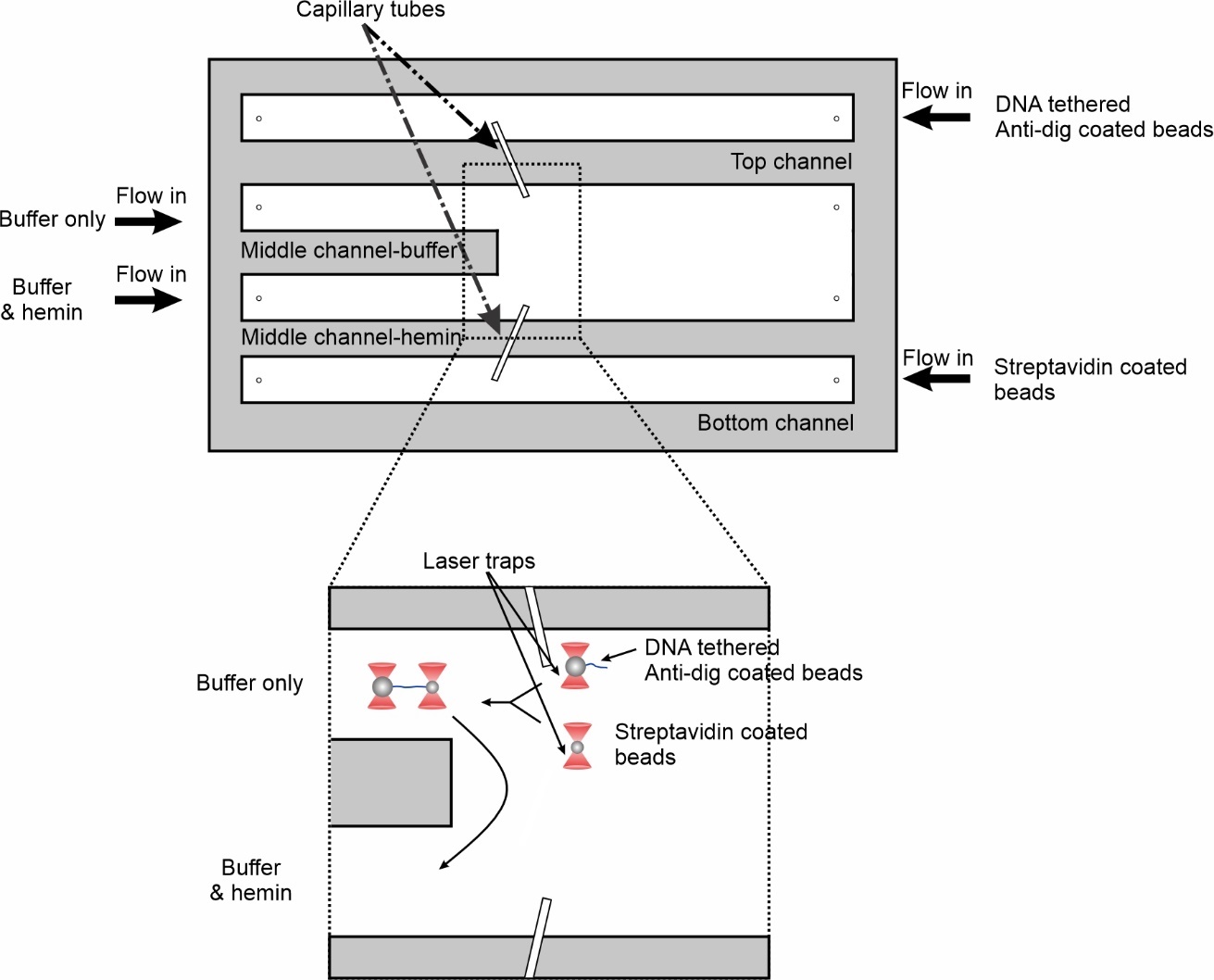
**

**Figure S10.** Schematic of a four-channel microfluidic chamber for optical-tweezers experiments.

**Results of optical-tweezers experiments**

We used optical tweezers instrument as described previously.^[^[^13^](#_ENREF_13)^]^ Briefly, A 1064 nm laser (4 W, continuous-wave mode, BL-106C, Spectra-physics) was split into two polarized beams to create two optical traps separately. The positions of both traps were independently tracked with position-sensitive photodetectors (PSD; DL100, Pacific Silicon Sensor). One trap was standstill while the other trap was actively controlled using a steerable mirror (Nano-MTA, Mad City Labs, Madison, WI).

To initiate the experiment, DNA samples were mixed with anti-digoxigenin-coated beads (~2.3 µm, Spherotech, Lake Forest, IL, USA) and introduced into the top channel, while streptavidin-coated beads (~1.8 µm, Spherotech, Lake Forest, IL, USA) were added to the bottom channel (Figure S10). Beads were transferred to the middle channel via capillary tubes (King Precision Glass, Inc., PO MC 7701), trapped by two laser foci, and brought together to tether a single DNA molecule. The construct was then subjected to force-ramping for single-molecule unfolding and folding measurements according to established protocols from published references.^[^[^22-23^](#_ENREF_22)^]^

During force-ramping, the tethered DNA molecule was extended (unfolding process) or relaxed (refolding process) by moving one bead away from or towards the other, respectively. The experiments were first performed in a buffer-only microchannel and then moved to an adjacent microchannel containing 25 mM hemin in the sucrose buffer (40% (w/v) sucrose, 100 mM KCl, 10 mM Tris, pH 7.4). Force–extension (F–X) curves were captured and processed using LabVIEW software (National Instruments, TX) during the procedure. Following each full stretching–relaxation cycle, the optical traps were held static at 0 pN for about 30 seconds to 2 minutes to enable the formation of DNA secondary structures. The force-ramping experiments were performed at 25 °C.

The WT Tel-4G GQ exhibited a rupture force of 17.5 pN (Figure S11A) and the change-in-contour-length (Δ*L*) of 8.9 nm (Figure S12, see “Expected Δ*L* calculations” below for detailed calculation), which aligns well with the expected Δ*L* for GQ structures (7.4–9.0 ± 0.1 nm)^[^[^16^](#_ENREF_16)^]^ and confirms the GQ formation. The GQ-11oxoG sequence formed GQs with a rupture force of 21.0 pN (Figure S11D) and a Δ*L* of 8.9 nm (Figure S13), consistent with expected GQ Δ*L* values. Despite its slightly higher mechanical strength compared to WT Tel-4G, the GQ-11oxoG sequence showed lower thermodynamic stability (58.1% formation, see Table S3 & “Calculation of the structural formation percentage” below for detailed calculation) than WT Tel-4G (80.5% formation). To systematically evaluate and compare stabilities, we introduced "weighted rupture force averages,"^[^[^24^](#_ENREF_24)^]^ which integrates mechanical and thermodynamic stabilities (Table S4, see “Calculation of weighted unfolding force average” for detailed calculation). This analysis revealed a greater overall stability for WT Tel-4G GQ (15.4 pN) compared to GQ-11oxoG (12.6 pN), consistent with UV melting results (Table S4, Figure S2).

Next, we investigated the stability of GQ-hemin complexes. At 25 µM hemin, the WT Tel-4G sequence displayed two populations (Figure S11B): one resembling the GQ-only state (16.7 pN, Δ*L* = 8.0 nm, see “Deconvoluted rupture force histograms” in Figure S15 for deconvolution of subpopulations) and another at 27.4 pN (Δ*L* = 8.0 nm), attributed to the GQ-hemin structure. Extended incubation time from 30 seconds to 2 minutes at 0 pN increased the GQ-hemin population (the 27.4 pN species) from 10% to 23% (Table S5, see “The weights of different populations” below for detailed calculation), confirming the GQ-hemin formation. The increased rupture force for the GQ-hemin was consistent with melting temperature measurements (Figures S2 & S14, *T*_m_ = 59.9 ± 0.5 °C for the GQ-hemin while *T*_m_ = 56.9 ± 0.6 °C for the WT GQ only). For the GQ-11oxoG sequence with hemin (Figure S11E), two populations were observed: one at 10.3 pN (Δ*L* = 7.5 nm, see “Deconvoluted rupture force histograms” in Figure S16 for deconvolution of subpopulations), assigned to the GQ-hemin, and another at 21.6 pN (Δ*L* = 7.5 nm), resembling the GQ-only state. Longer incubation time (from 30 seconds to 2 minutes at 0 pN) raised the fraction of the lower-force GQ-11oxoG-hemin from 21% to 37% (Table S5), further validating the GQ-11oxoG-hemin assignment. The decreased rupture force for the GQ-11oxoG-hemin was consistent with melting temperature measurements (Figure S14, *T*_m_=11.5 ± 6.4 °C for the GQ-11oxoG-hemin while *T*_m_ = 25.1 ± 0.1 °C for the GQ-11-oxoG only). Overall, WT Tel-4G GQ-hemin exhibited significantly higher stability (~26 pN, Figure S15) than the GQ-11oxoG-hemin (~10 pN, Figure S16), a trend consistent with weighted rupture force averages (see “Calculation of weighted unfolding force average” for the calculation. WT Tel-4G GQ-hemin: 22.9 pN; GQ-11oxoG-hemin: 6.6 pN; Table S4). In addition, their thermodynamic stabilities also reflect this trend (*T*_m_ = 59.9 ± 0.5 °C for the WT-GQ-hemin vs *T*_m_ = 11.5 ± 6.4 °C for GQ-11oxoG-hemin). It is significant that the GQ-11oxoG bound with hemin has reduced stabilities compared to the free GQ-11oxoG. This result is consistent with previous findings^[^[^25^](#_ENREF_25)^]^ that some ligands can destabilize GQ structures.


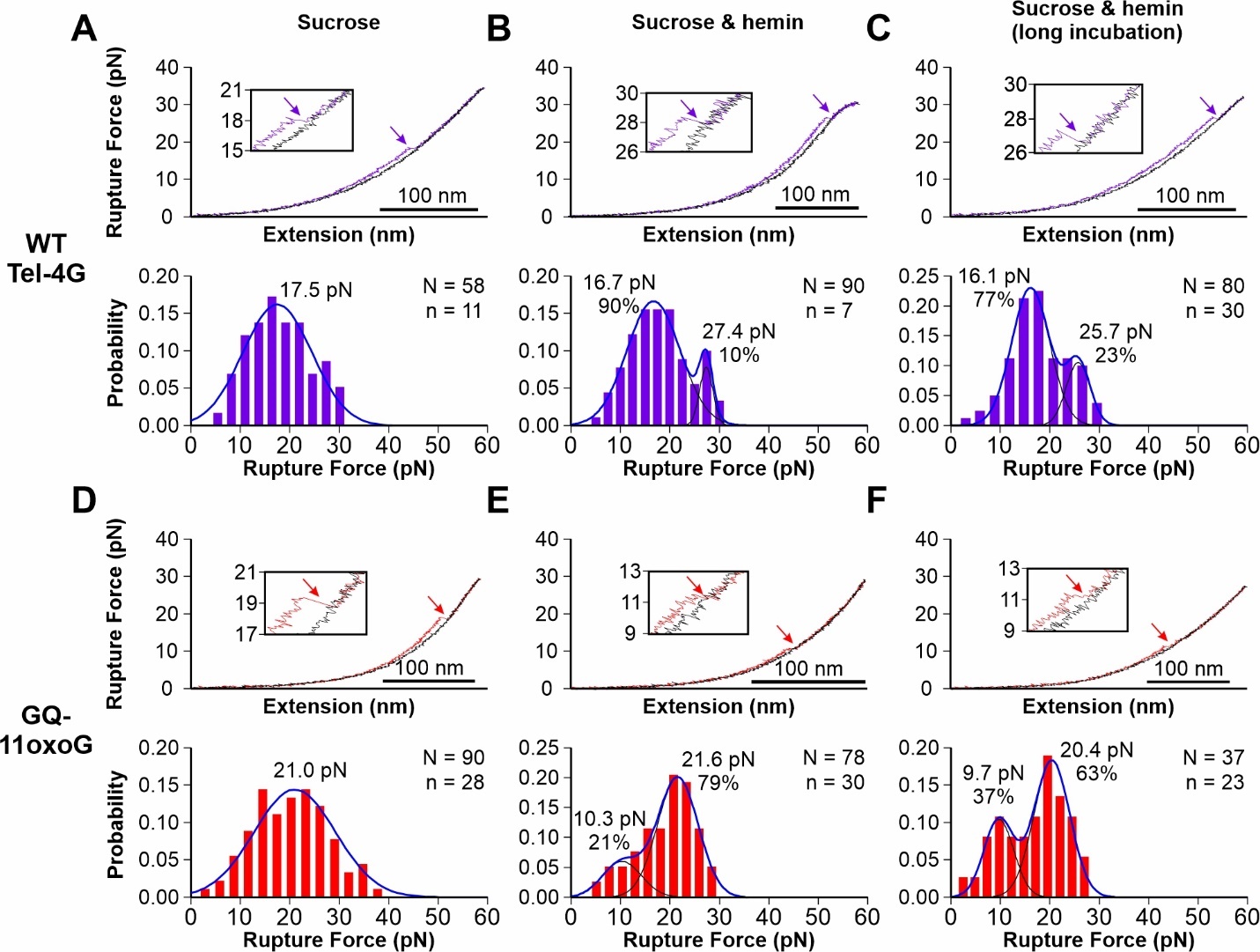


**Figure S11.** Mechanical stabilities of WT Tel-4G and GQ-11oxoG GQs revealed by single-molecule mechanical unfolding experiments in optical tweezers. A) & D) Representative force-extension (F-X) curves and rupture force histograms for WT Tel-4G and GQ-11oxoG in sucrose buffer (40% (w/v) sucrose, 100 mM KCl, 10 mM Tris, pH 7.4) with a 30-second incubation time. B) & E) F-X curves and rupture force histograms for WT Tel-4G and GQ-11oxoG in the presence of 25 µM hemin in the same sucrose buffer under a 30-second incubation time. C) & F) F-X curves and rupture force histograms for WT Tel-4G and GQ-11oxoG with 25 µM hemin in the sucrose buffer under a 2-minute incubation time. All experiments were conducted at 25°C. Here, N denotes the number of features, and n represents the number of molecules analyzed. Arrows indicate rupture events.


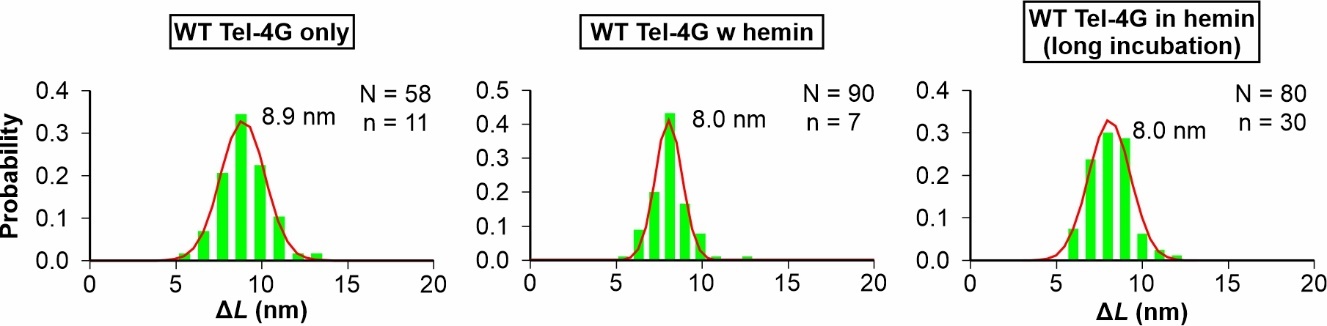


**Figure S12.** The Δ*L* histograms of the WT Tel-4G sequence obtained in the optical-tweezers experiments in sucrose buffer without (left) and with (middle) 25 µM hemin under normal incubation time (30 seconds) or with long incubation time (2 minutes, right). Sucrose buffer contains a 10 mM Tris buffer in 40% (w/v) sucrose supplemented with 100 mM KCl, pH 7.4. Solid curves depict Gaussian fittings. N and n represent the numbers of features and molecules, respectively.


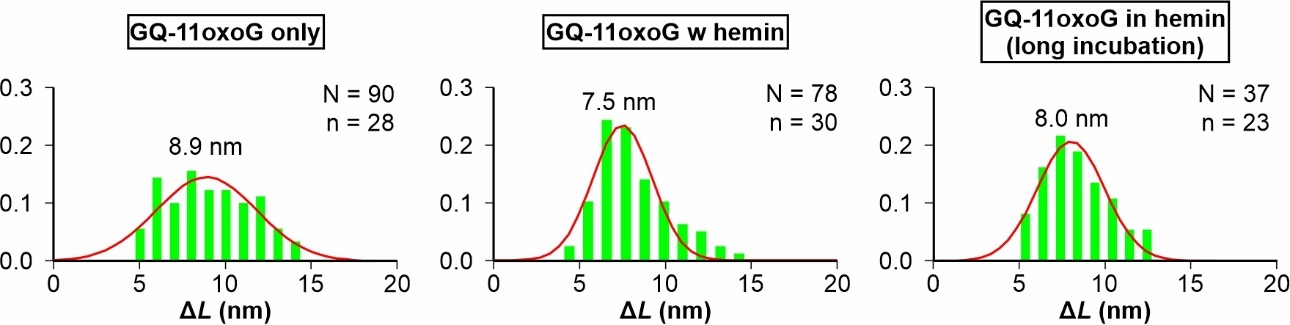


**Figure S13.** The Δ*L* histograms of the GQ-11oxoG sequence obtained in the optical-tweezers experiments in sucrose buffer without (left) and with (middle) 25 µM hemin under normal incubation time (30 seconds) or with long incubation time (2 minutes, right). Sucrose buffer contains a 10 mM Tris buffer in 40% (w/v) sucrose supplemented with 100 mM KCl, pH 7.4. Solid curves depict Gaussian fittings. N and n represent the numbers of features and molecules, respectively.

**Expected Δ*L* calculations used in the optical-tweezers experiments**

The expected change-in-contour-length (Δ*L*) is calculated based on the Equation S1,

$\Delta L=\left( N\times L_{\mathrm{nt}} \right)-x$ (Equation S1),

here, *N* represents the number of nucleotides in the DNA molecule, *L*ₙₜ denotes the average contour length per nucleotide (ranging from 0.40 to 0.48 nm/nt),^[^[^23^](#_ENREF_23)^,^ [^26-28^](#_ENREF_26)^]^ and *x* refers to the end-to-end distance of the folded DNA secondary structure.

For G-quadruplexes (GQs), the number of nucleotides (*N*) is typically 21, while the end-to-end distance of the folded DNA secondary structure (*x*) varies based on specific GQ conformations. For instance, *x* is approximately 1.1 ± 0.1 nm for hybrid-1 type GQs^[^[^29^](#_ENREF_29)^]^ and 1.8 ± 0.1 nm for basket-type GQs^[^[^30^](#_ENREF_30)^]^. Consequently, the expected change-in-contour-length (Δ*L*) for GQs ranges from approximately 7.4 to 9.0 ± 0.1 nm.^[^[^16^](#_ENREF_16)^]^

In this study, both WT Tel-4G GQ and GQ-11oxoG sequences exhibited an expected Δ*L* of 7.4 to 9.0 ± 0.1 nm for G-quadruplex structures. Experimental measurements for the WT Tel-4G GQ sequence showed a Δ*L* range of 8.0 to 8.9 nm (Figure S12), consistent with the expected range and confirming GQ formation. Similarly, the GQ-11oxoG sequence displayed a Δ*L* range of 7.5 to 8.9 nm (Figure S13), supporting the formation of GQ structures.

**Calculation of the structural formation percentage in the optical-tweezers experiments**

The structural formation percentage (Formation%) was used as a measurement of the thermodynamic stability of WT Tel-4G GQ and GQ-11oxoG (Table S3).^[^[^24^](#_ENREF_24)^]^ It was calculated using the number F-X traces that contained unfolding features vs total number of F-X traces (Equation S2):

*Formation%* $=\begin{matrix} \frac{Number of curves with unfolding features}{Number of all curves}\times100\% & \end{matrix}$(Equation S2).

**Table S3.** Formation% of WT Tel-4G and GQ-11oxoG in the sucrose buffer (40 % (w/v) sucrose, 100 mM KCl, 10 mM Tris, pH 7.4).

| **Sample** | **Formation%** |
| --- | --- |
| WT Tel-4G GQ | 80.5 |
| GQ-11oxoG | 58.1 |

**Calculation of weighted unfolding force average obtained in the optical-tweezers experiments**

The weighted unfolding force average^[^[^24^](#_ENREF_24)^]^ was used to estimate the overall mechanical stability of secondary structures formed in a given DNA sequence:

*Weighted unfolding force average* $=\begin{matrix} \frac{\sum(Unfolding force in a particular bin bin size)}{Number of data points} & \end{matrix}$

(Equation S3).

## The bin was determined from the unfolding force histograms (see Figure S11). For a force-extension (F-X) curve lacking unfolding features indicative of folded structures, an unfolding force of 0 pN was assigned to each curve and included as a single data point in the analysis. The weighted unfolding force averages (pN) for G-quadruplexes (GQs) and their corresponding GQ-hemin complexes are presented in Table S4.

**Table S4.** Weighted unfolding force averages (pN) of WT Tel-4G or GQ-11oxoG without and with hemins.

| **Sample** | **Weighted unfolding force averages (pN)** |
| --- | --- |
| WT Tel-4G GQ | 15.4 |
|  |  |
| WT Tel-4G GQ-hemin | 22.9 |
|  |  |
| GQ-11oxoG | 12.6 |
|  |  |
| GQ-11oxoG-hemin | 6.6 |

**The weights of different populations** **obtained in the optical-tweezers experiments**

Table S5 shows the weights of different populations under different conditions. The weights calculation is based on the following equation:

*Weights* $=\begin{matrix} \frac{Number of data points in one population}{Number of all data points} & \end{matrix}$

(Equation S4).

**Table S5.** Weights of different populations under different conditions.

| **Name** | **Conditions** | **Populations**  **(rupture force/Δ*L*)** | **Weights** |
| --- | --- | --- | --- |
| WT Tel-4G | Sucrose  (30 sec incubation) | GQ  (17.5 pN/8.9 nm) | 100% |
|  |  |  |  |
|  | Sucrose with hemin  (30 sec incubation) | GQ  (16.7 pN/8.0 nm) | 90% |
|  |  | GQ-hemin complex  (27.4 pN/8.0 nm) | 10% |
|  | Sucrose with hemin  (2 min incubation) | GQ  (16.1 pN/8.0 nm) | 77% |
|  |  | GQ-hemin complex  (25.7 pN/8.0 nm) | 23% |
|  |  |  |  |
| GQ-11oxoG | Sucrose  (30 sec incubation) | GQ  (21.0 pN/8.9 nm) | 100% |
|  |  |  |  |
|  | Sucrose with hemin  (30 sec incubation) | GQ-hemin complex  (10.3 pN/7.5 nm) | 21% |
|  |  | GQ  (21.6 pN/7.5 nm) | 79% |
|  | Sucrose with hemin  (2 min incubation) | GQ-hemin complex  (9.7 pN/8.0 nm) | 37% |
|  |  | GQ  (20.4 pN/8.0 nm) | 63% |

**
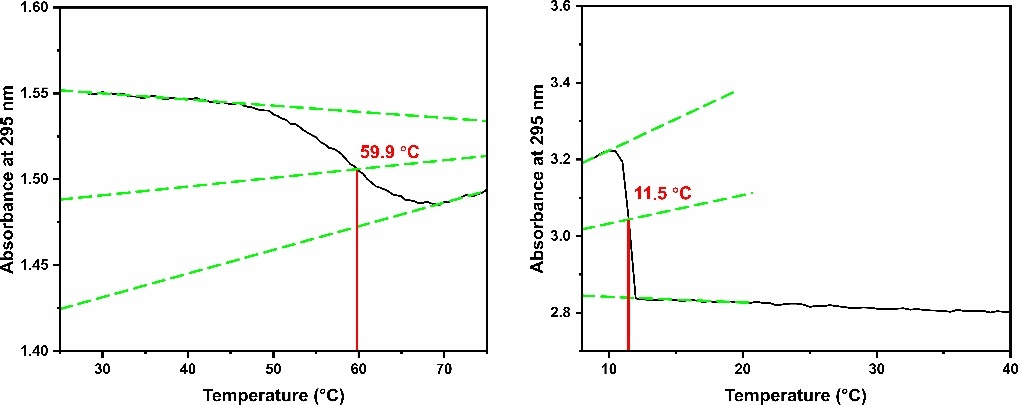
**

**Figure S14.** Typical UV melting curves of GQ–hemin complexes. Left, 5 µM WT Tel-4G mixed with 100 µM hemin. Right, 5 µM GQ-11oxoG mixed with 25 µM hemin. The reason 25 µM instead of 100 µM hemin was used in GQ-11oxoG–hemin is because hemin destabilizes GQ-11oxoG (see “Results of optical-tweezers experiments” for details). Therefore, a high hemin concentration (100 µM) led to complete disruption of the GQ-11oxoG structure, resulting in the absence of a detectable 295-nm absorbance signal. Samples were prepared in sucrose buffer (40 % (w/v) sucrose, 100 mM KCl, 10 mM Tris, pH 7.4). UV melting was performed at 295 nm in a Cary 300 spectrophotometer (Agilent Technologies, USA) with a temperature ramp rate of 0.5 °C/min. The *T*_m_ of the WT Tel-4G GQ-hemin complex was 59.9 ± 0.5 °C, higher than that of the WT Tel-4G GQ alone (56.9 ± 0.6 °C), whereas the *T*_m_ of the GQ-11oxoG-hemin complex was 11.5 ± 6.4 °C, substantially lower than that of GQ-11oxoG alone (25.1 ± 0.1 °C).

**Deconvoluted rupture force histograms** **obtained in the optical-tweezers experiments**

To account for the stochastic variability observed in individual unfolding events, rupture force events at the intersection of two adjacent Gaussian populations were randomly assigned to one of the two populations. The assignment probability was weighted based on the relative proportion of each population within the corresponding bin of the interaction.^[^[^24^](#_ENREF_24)^]^ The deconvoluted rupture force histograms (Figures S15 and S16) enabled assignment of these populations to distinct folded structural states (see “Results of optical-tweezers experiments” for details).


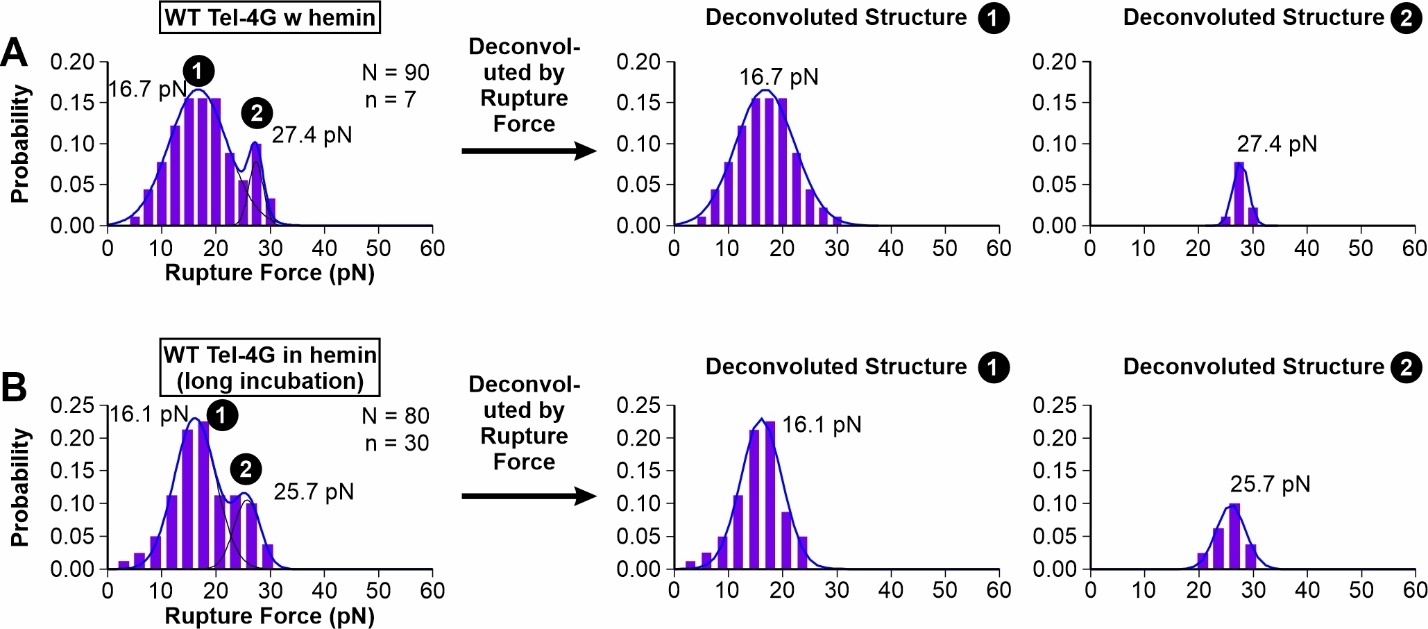


**Figure S15.** Deconvoluted rupture force histograms of the WT Tel-4G sequence obtained in the optical-tweezers experiments in hemin & sucrose buffer with normal incubation time (30 seconds, A) and with long incubation time (2 minutes, B). Hemin & sucrose buffer contains a 10 mM Tris buffer in 40% (w/v) sucrose supplemented with 25 µM hemin and 100 mM KCl, pH 7.4. Solid curves depict Gaussian fittings. N and n represent the numbers of features and molecules, respectively.

**
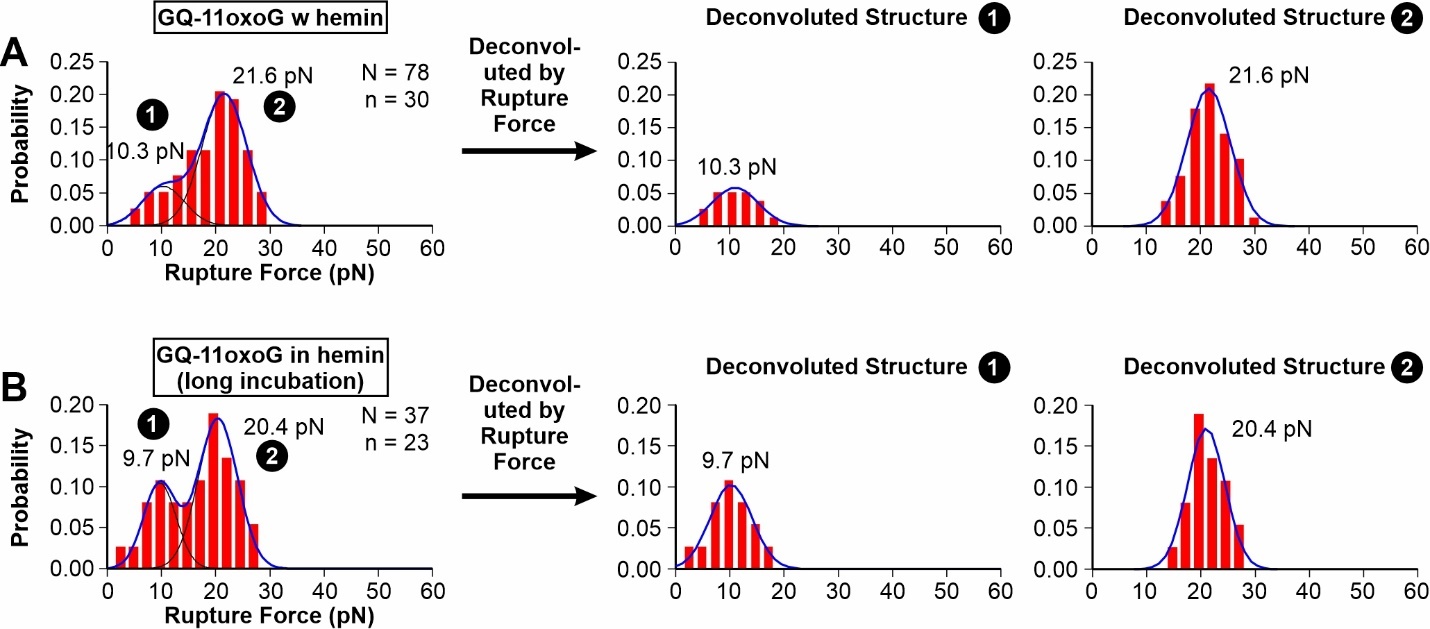
**

**Figure S16.** Deconvoluted rupture force histograms of the GQ-11oxoG sequence obtained in the optical-tweezers experiments in hemin & sucrose buffer with normal incubation time (30 seconds, A) and with long incubation time (2 minutes, B). Hemin & sucrose buffer contains a 10 mM Tris buffer in 40% (w/v) sucrose supplemented with 25 µM hemin and 100 mM KCl, pH 7.4. Solid curves depict Gaussian fittings. N and n represent the numbers of features and molecules, respectively.

## S8. Fluorescence bulk experiments under ultrasonication

To measure the turnover activities of the AR🡪RF reactions (Figure 4) by the GQ-hemin complexes under ultrasonication, the probe of a Digital Sonifier 450 (frequency 10 kHz, Branson Ultrasonics Corporation) was inserted into the outer compartment of a modified container filled with deionized (DI) water (Figure S17). The DI water served as a medium to transmit ultrasonication energy to the inner sample holder containing the reaction buffer. The temperature of the water bath was maintained at 25 °C by manual water exchange. The values of initial velocity (*V*_0_) are presented in Figures S18 & S19 and Table S6, with both exponential and linear fitting results exhibiting consistent trends.

**
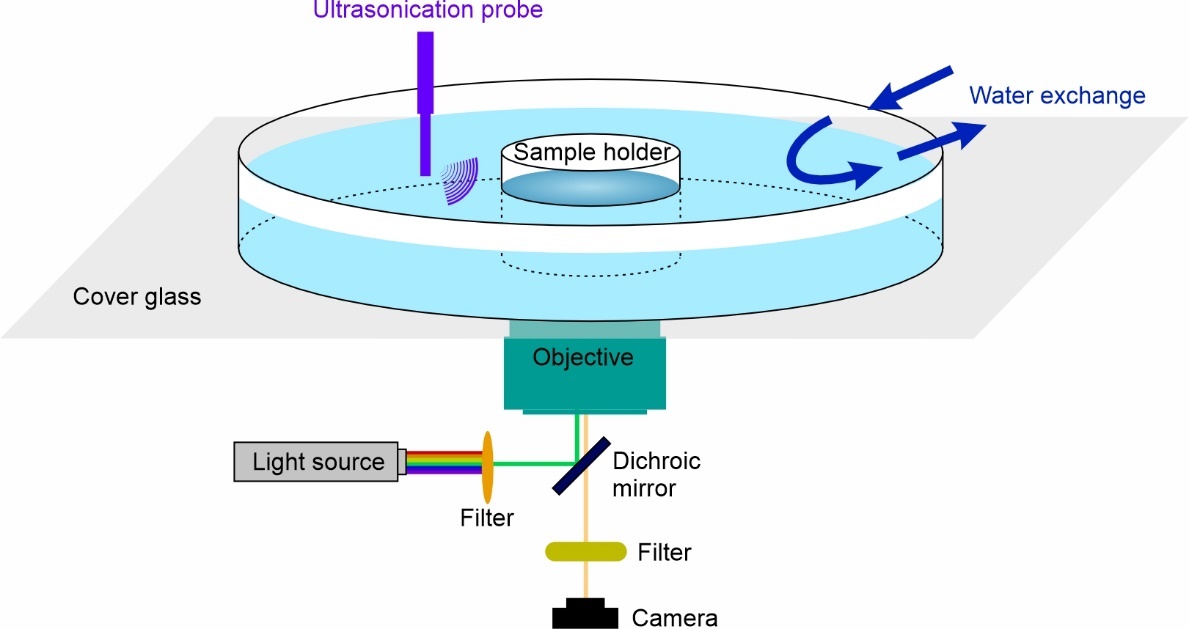
**

**Figure S17.** Setup details of the GQ-hemin catalyzed AR🡪RF reaction (see Figure 4A) inside the sample holder under ultrasonication.

**Measurements of ultrasonication powers**

We used a customized hydrophone (a sourced piezoelectric ceramic discs (Cheerock, Amazon) composed of lead zirconate titanate) to measure ultrasonication powers during experiments.^[^[^31^](#_ENREF_31)^]^ First, the customized hydrophone converted ultrasonication signals into oscillating voltage signals (*V_i_*) via a digital oscilloscope (Hantek 6022BE, 20 MHz bandwidth, 48 MSa/s sampling rate), which was further calculated to obtain the RMS voltage (*V*_RMS_)^[^[^32^](#_ENREF_32)^]^:

$V_{RMS}=\sqrt{\frac{1}{N}\sum_{i=1}^{N} V_{i}^{2}}$ (Equation S5),

here *V*_RMS_ is the statistical quantification representing the mean amplitude of an oscillating voltage signal over a 30-second interval, during which the ultrasound device remained continuously on.

With *V*_RMS_ and equations S5-S7, the acoustic intensity (ultrasound power) (*I*) can be calculated as,

$P= \frac{V_{RMS}}{M}$ (Equation S6),

$I=\frac{P^{2}}{2 c}$ (Equation S7),

*P*: the acoustic pressure (unit: Pa).

*M*: the sensitivity of the customed hydrophone (unit: V/Pa).

*I*: the acoustic intensity or ultrasound power (unit: W/m²).

*ρ*: the density of the medium (water) (unit: kg/m³).

c: the speed of sound in medium (water) (unit: m/s).

To ensure data accuracy, the sensitivity of the customized hydrophone (*M*) was calibrated against the commercial reference hydrophone (model H1a, Aquarian Audio) under the same experimental condition, in accordance with the IEC 60565-1:2020 standard.

Finally, the effective ultrasound power (*P*_eff_) employed in the experiments (Figure 4B&C) was quantified by the measured ultrasound power (*I*) and the specific duty cycle with the Equation S8:

$P_{eff}=I\times DC$ (Equation S8),

where *I* denotes the acoustic intensity or ultrasound power (unit: W/m²) (Equation S7), and DC (duty cycle) represents the percentage of ultrasound on-time, calculated as $DC=$ (pulse-on duration / (pulse-on duration + pulse-off duration)) ×100%.


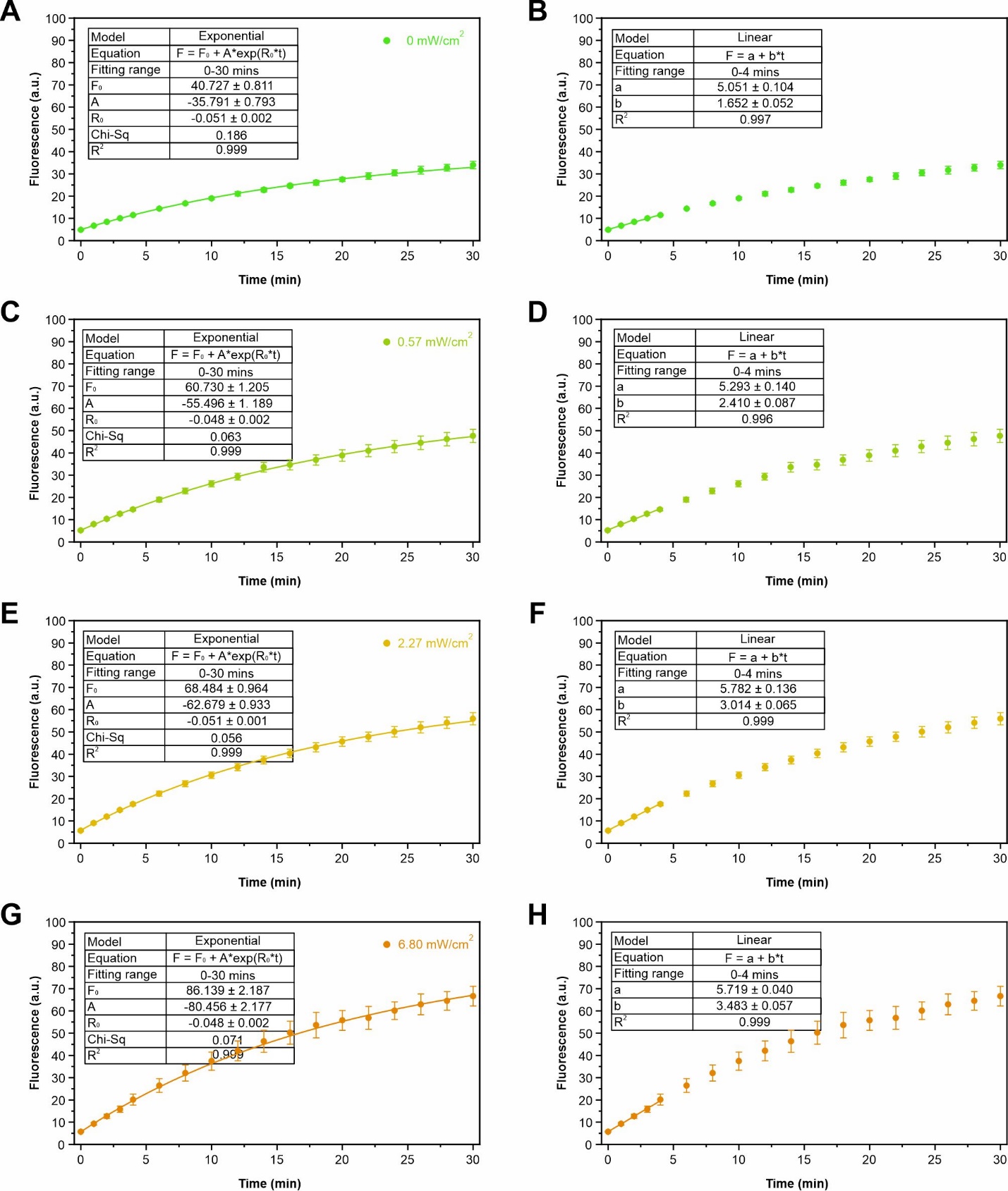


**Figure S18.** Fluorescence intensity versus time curves of the GQ-11C-hemin catalyzed AR🡪RF reaction (see Figure 4) at 0 mW/cm² (A, B), 0.57 mW/cm² (C, D), 2.27 mW/cm² (E, F), and 6.80 mW/cm² (G, H). The curves were fitted with exponential (left panels) or linear (right panels) equations.


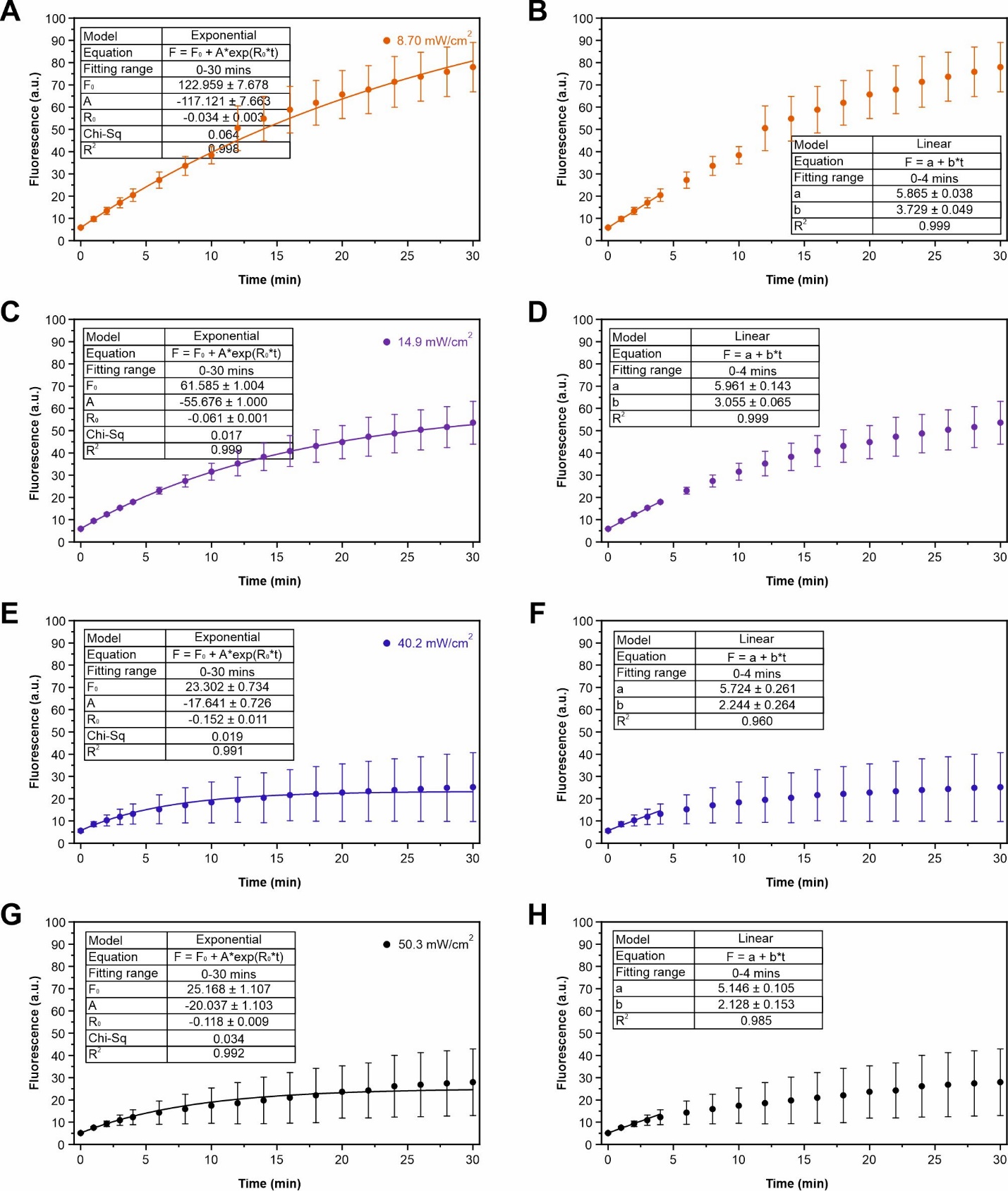


**Figure S19.** Fluorescence intensity versus time curves of the GQ-11C-hemin catalyzed AR🡪RF reaction (see Figure 4) at 8.70 mW/cm² (A, B), 14.9 mW/cm² (C, D), 40.2 mW/cm² (E, F), and 50.3 mW/cm² (G, H) were fitted with an exponential (left panels) or a linear (right panels) equation.

**Table S6.** Initial velocity (*V*_0_, (a.u.)/min) of GQ-11C-hemin at different ultrasonication powers (see Figure 4D).

| **Ultrasonication power (mW/cm^2^)** | ***V*_0_**  **(Exponential fitting)** | ***V*_0_**  **(Linear fitting)** |
| --- | --- | --- |
| 0 | 1.82 ± 0.07 | 1.65 ± 0.05 |
| 0.57 | 2.64 ± 0.10 | 2.41 ± 0.09 |
| 2.27 | 3.20 ± 0.09 | 3.01 ± 0.07 |
| 6.80 | 3.86 ± 0.20 | 3.48 ± 0.06 |
| 8.70 | 3.99 ± 0.43 | 3.73 ± 0.05 |
| 14.9 | 3.41 ± 0.10 | 3.06 ± 0.07 |
| 40.2 | 2.68 ± 0.23 | 2.24 ± 0.26 |
| 50.3 | 2.37 ± 0.22 | 2.13 ± 0.15 |

To investigate whether the observed decrease in fluorescence intensity resulted from bond cleavage in the GQ-11C DNA, which would disrupt the GQ-hemin complex, we conducted electrophoresis using 20% denaturing PAGE to quantify intact GQ-11C after sono-mechanical experiments (Figure S20A). A 48-nt random-sequence single-stranded DNA (see Table S1 for the sequence, “Random ssDNA for internal standard”) was included as an internal standard to account for loading errors. The band-intensity ratio of GQ-11C to the internal standard was analyzed to evaluate potential strand cleavage. The results (Figure S20 B&C) revealed negligible GQ-11C degradation across all ultrasound conditions tested, confirming that the fluorescence reduction was not due to ultrasonication-induced cleavage in GQ-11C.

**
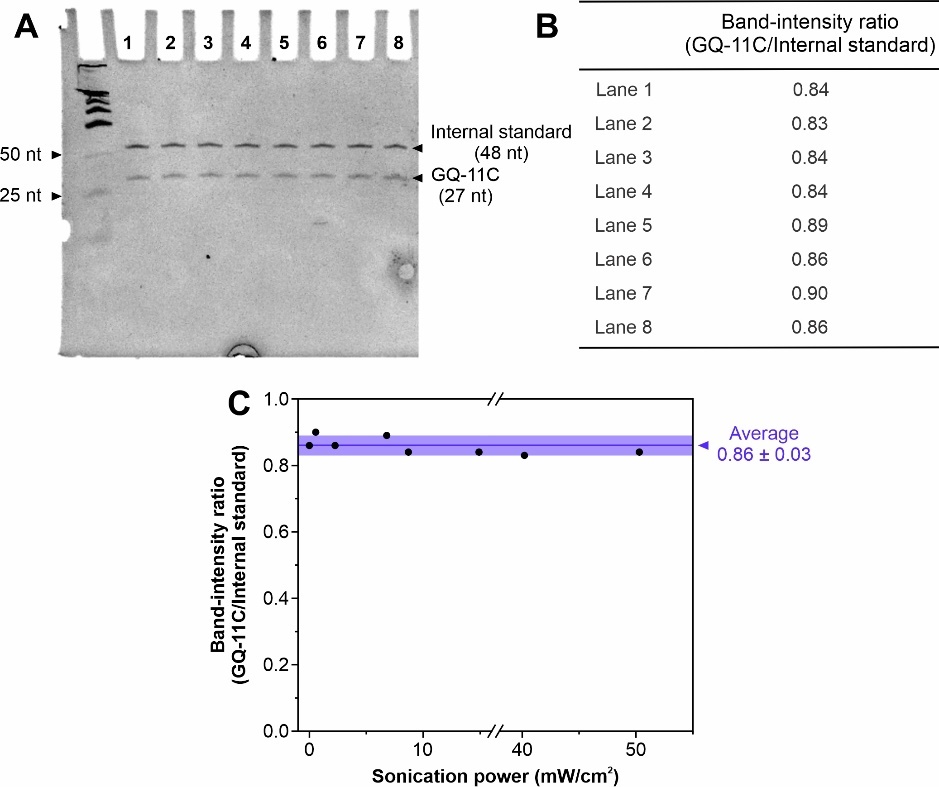
**

**Figure S20.** The PAGE gel image (A) of the GQ-11C sequence after sono-mechanical experiments (Figures S17-19). (Lane 1: sample after 50.3 mW/cm^2^ ultrasonication, Lane 2: sample after 40.2 mW/cm^2^ ultrasonication, Lane 3: sample after 14.9 mW/cm^2^ ultrasonication, Lane 4: sample after 8.70 mW/cm^2^ ultrasonication, Lane 5: sample after 6.80 mW/cm^2^ ultrasonication, Lane 6: sample after 2.27 mW/cm^2^ ultrasonication, Lane 7: sample after 0.57 mW/cm^2^ ultrasonication, Lane 8: sample without ultrasonication). Since the band-intensity ratio shows only slight fluctuations (average 0.86 ± 0.03, all values are not significantly deviated from the average (C) at 95 % confidence level) across different ultrasonication powers (0–50.3 mW/cm^2^), it demonstrates that the GQ-11C strand remains intact, indicating no obvious strand cleavage (B).

**Activity recovery of DNAzyme after ultrasonic treatment**

To evaluate whether the DNAzyme activity can be recovered after ultrasonication, the DNAzyme sample (25 µM, GQ-11C and hemin at a 1:1 ratio in sucrose buffer containing 40% (w/v) sucrose, 100 mM KCl, and 10 mM Tris, pH 7.4) was first subjected to ultrasound at the power density of 50.3 mW/cm² (frequency 10 kHz) for 30 minutes at 25 °C. The sample was then incubated at 4 °C for 3 hours to allow structural recovery. Subsequently, the reaction mixture (0.25 µM GQ-11C-hemin, 0.5 mM H₂O₂, and 10 µM AR) was prepared and analyzed following the procedure described in Supporting Information Section 1. The initial velocity (*V*_0_, (a.u.)/min) was determined from the fluorescence intensity versus time curves.

The initial velocities (*V*_0_, (a.u.)/min) of the DNAzyme without ultrasonication (1.82 ± 0.07) and after recovery from ultrasonication (2.01 ± 0.30) are identical within experimental error (*p*=0.34, Figure S21), indicating that the DNAzyme activity was not affected by ultrasonication.

In addition, electrophoretic analysis using 20% denaturing PAGE further confirmed that GQ-11C underwent negligible degradation after the ultrasonic treatment (Figure S22).


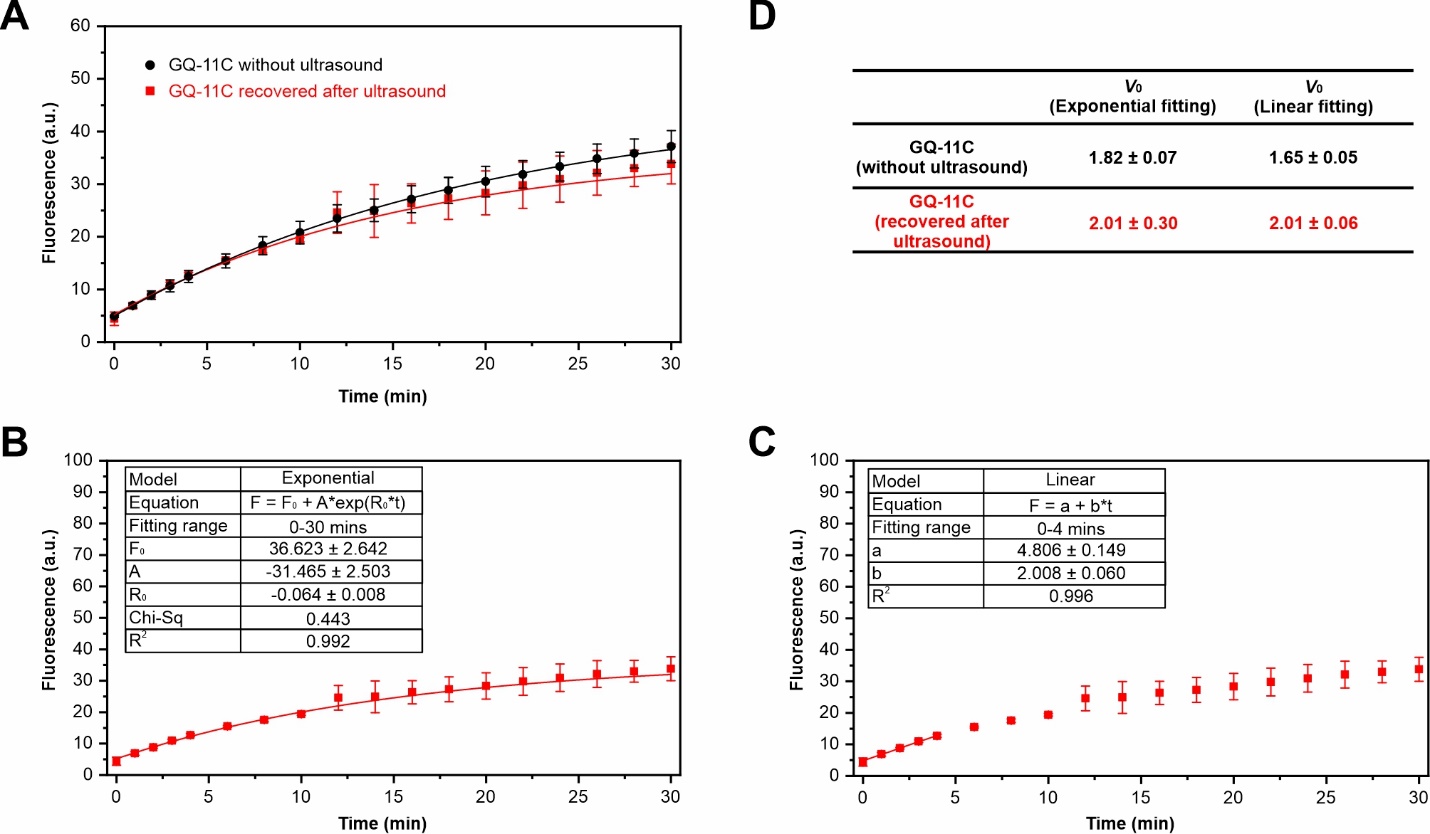


**Figure S21.** (A) Fluorescence intensity versus time curves of the catalysis (AR🡪RF reaction at 25 °C without ultrasonication) of the GQ-11C-hemin (see Figure 4) before ultrasonication (black) and recovered after the ultrasonication (red). The data points were fitted with an exponential equation (solid curves). The GQ-11C-hemin after the ultrasonication was recovered as the following: 25 µM GQ-11C-hemin solution was ultrasonicated under 50.3 mW/cm² power (frequency 10 kHz) for 30 minutes and then placed in 4°C for 3 hours. (B, C) were the fluorescence intensity versus time curves of the recovered GQ-11C-hemin without ultrasonication. The curves were fitted with an exponential (left panel) or a linear (right panel) equation. (D) Initial velocities (*V*_0_, (a.u.)/min) of the GQ-11C-hemin and the recovered GQ-11C-hemin.


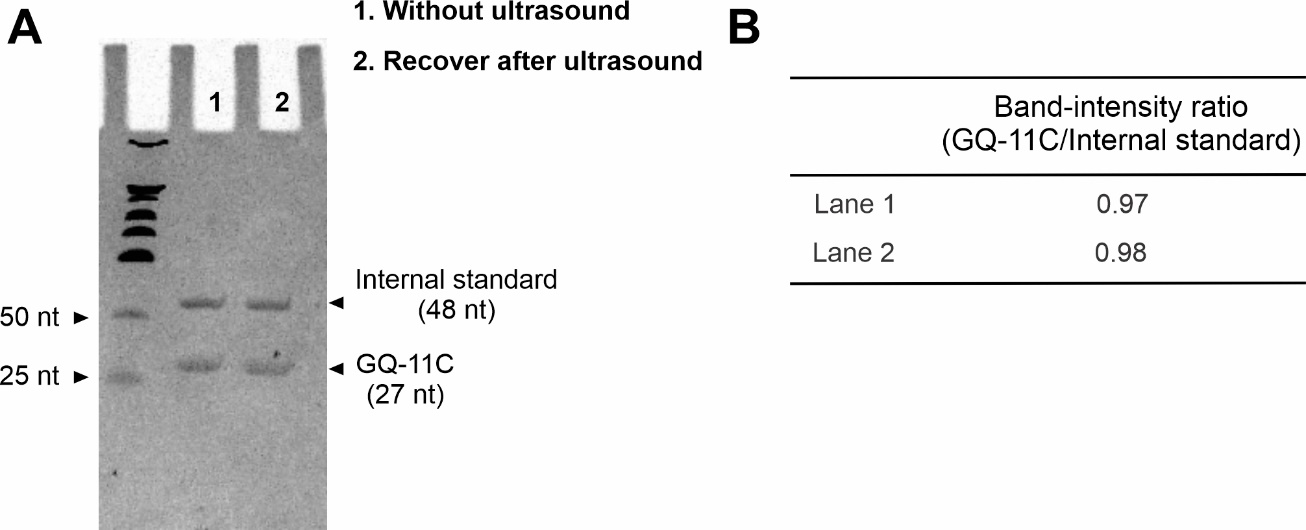


**Figure S22.** Determination of sono-cleavage of DNA samples. (A) A 20% denaturing PAGE gel image comparing the GQ-11C sample without ultrasonication and the GQ-11C sample recovered after exposure to the ultrasonication at an intensity of 50.3 mW/cm^2^ (frequency 10 kHz). A 48-nt random-sequence single-stranded DNA (see Table S1 for the sequence, “Random ssDNA for internal standard”) was included as the internal standard to account for loading errors. (B) Quantitative analysis of band intensities shows only a minor variation in the band-intensity ratio (0.97 for the untreated sample and 0.98 for the sample after ultrasonication). This negligible change indicates that the GQ-11C DNA remains intact, demonstrating that no detectable strand cleavage occurs under applied ultrasonication conditions.


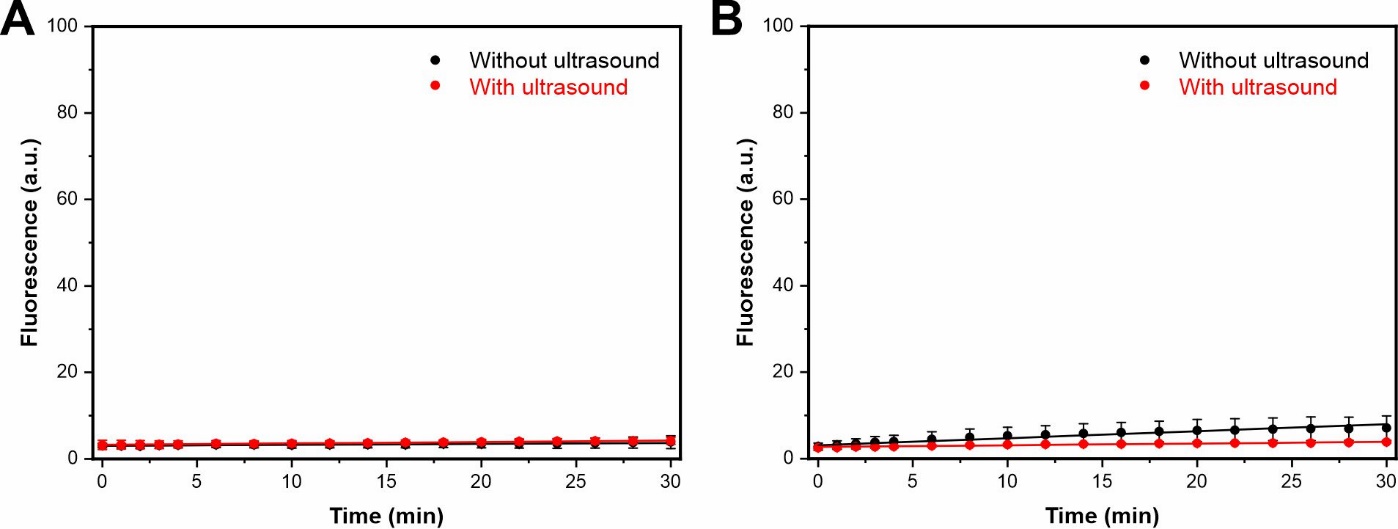


**Figure S23.** The fluorescence intensity versus time curves of the control experiments for Figure 4. The reaction sample in A: 10 µM AR and 0.5 mM H_2_O_2_ in the sucrose buffer (40% (w/v) Sucrose, 100 mM KCl, 10mM Tris at pH 7.4). The reaction sample in B: 0.25 µM hemin, 10 µM AR, and 0.5 mM H_2_O_2_ in the sucrose buffer (40% (w/v) Sucrose, 100 mM KCl, 10mM Tris at pH 7.4). Solid lines were fitted by the linear equation. The ultrasound power was 8.70 mW/cm^2^ and the reactions were performed at 25 °C. These experiments indicated that neither ultrasonication-generated radicals nor the hemin released from the GQ-hemin complex made a significant contribution to the enhanced catalytic activity observed in Figure 4B.

## S9. Sono-melting experiments

The sono-melting experimental setup was adapted from the design described in Figure S17. Instead of using an ultrasonication probe, an ultrasonic transducer (FS30 sonic cleaner, Thermo Scientific) was used. The 200 µM Thioflavin T (ThT), which fluoresces (excitation ~425 nm, emission ~490 nm) upon binding to DNA G-quadruplexes (GQs),^[^[^33^](#_ENREF_33)^]^ was added to a 100 µL GQ–ThT solution (12.5 µM GQ-11C). This solution was subjected to thermal melting (2 °C to 60 °C at 1 °C/min) while the temperature was controlled by a PID temperature controller (Model: SYL-2352P, Amazon). The ultrasonication was applied to the solution mixture at 460 mW/cm^2^ throughout the entire melting process of 59 minutes. Signals of quenched fluorescence due to ThT released from melted GQ were recorded on an inverted fluorescence microscope (Nikon TE2000-U). Changes in fluorescence intensity indicated GQ unfolding and refolding processes, reflecting GQ thermostability with and without ultrasound (Figure S24). Figure S25 shows PAGE evidence that the fluorescence changes induced by ultrasound arose from the structural alterations of the GQ rather than cleavage of the GQ containing DNA molecules.


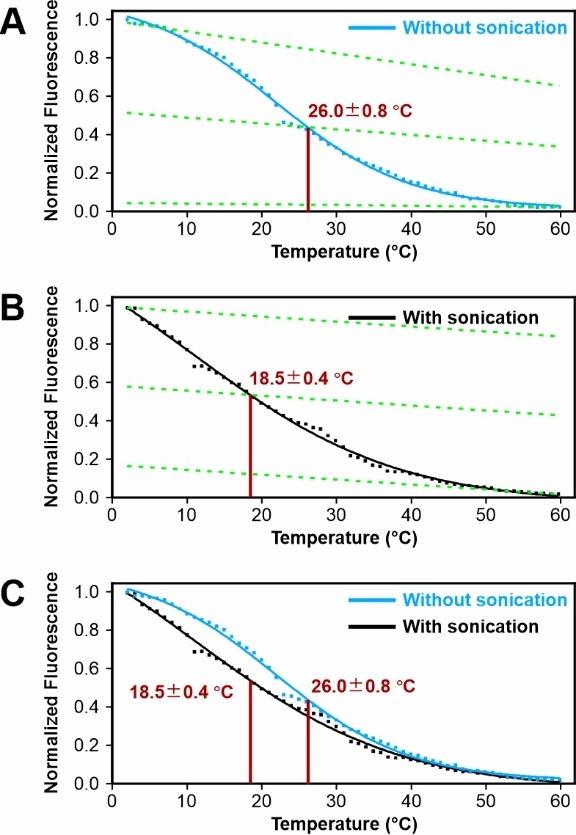


**Figure S24.** The fluorescence thermal melting curves of the GQ-11C without (A, blue) and with ultrasound (B, black) (460 mW/cm^2^) in sucrose buffer (40% (w/v) sucrose, 100 mM KCl, 10 mM phosphate, pH 7.4). C) The overlapped fluorescence melting curves of GQ-11C without and with ultrasound. *T*_m_ was determined using the methods described in Section S3. It is clear that under ultrasonication, the GQ-11C reduced thermal stabilities from *T*_m_=26.0 ± 0.8 °C to *T*_m_=18.5 ± 0.4 °C.

To determine whether the observed decrease in fluorescence intensity (Figure S24B) resulted from bond cleavage in the GQ-11C strand, which would disrupt the GQ–ThT and quench fluorescence, we performed electrophoresis using 20% denaturing PAGE to quantify intact GQ-11C strand after sono-melting experiments (Figure S25A). A 48-nt random-sequence single-stranded DNA (see Table S1 for the sequence “Random ssDNA for internal standard”) was used as an internal standard to correct for loading variability. The band-intensity ratio of GQ-11C to the internal standard was analyzed to assess potential strand cleavage. The results (Figure S25B) showed negligible GQ-11C degradation under the sono-melting condition (460 mW/cm^2^), confirming that the fluorescence reduction was not attributable to ultrasonication-induced cleavage in the GQ-11C molecule.


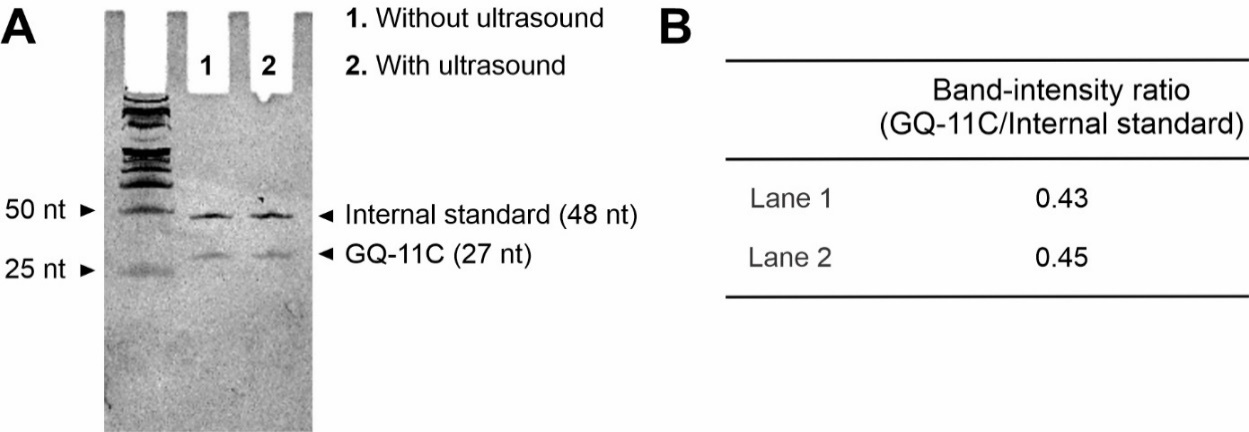


**Figure S25.** Determination of sono-cleavage of DNA samples. The PAGE gel image (A) comparing GQ-11C samples with and without 59-min ultrasonication exposure at 460 mW/cm². Since the band-intensity ratio shows slight fluctuations (0.43 without ultrasonication and 0.45 after ultrasonication), it demonstrates that the GQ-11C DNA remains intact (B), indicating no detectable strand cleavage under the ultrasonication.

## S10. Summary of enzyme activities and catalytic properties

## **Table S7.** Catalytic efficiencies of enzymes to oxidize AR🡪RF in presence of H_2_O_2_ (see Figure 1A for the reaction).

| **Catalyst** | ***k*_cat_/*K*_M_**  **(s^−1^μM^−1^)** | **Source** |
| --- | --- | --- |
| Hemin | 0.08 | ref^[^[^34^](#_ENREF_34)^]^ |
| 8 nm Fe_3_O_4_ NPs | 0.04 | ref^[^[^35^](#_ENREF_35)^]^ |
| 5 nm Pt NPs | 0.08 | ref^[^[^36^](#_ENREF_36)^]^ |
| 100nmSiO_2_@5 nm Pt NPs | 0.46 | ref^[^[^37^](#_ENREF_37)^]^ |
| 100nmSiO_2_@5 nm Pt NPs@120 nm mSiO_2_ | 1.60 | ref^[^[^37^](#_ENREF_37)^]^ |
| Coronzyme (AuNP@DNA) | 0.75 | ref^[^[^11^](#_ENREF_11)^]^ |
| Heme-Myoglobin Oligos (F10) | 0.12 | ref^[^[^34^](#_ENREF_34)^]^ |
| Heme-Myoglobin Oligos (F9) | 0.33 | ref^[^[^34^](#_ENREF_34)^]^ |
| Heme-Myoglobin Oligos (F1) | 0.63 | ref^[^[^34^](#_ENREF_34)^]^ |
| Heme-Myoglobin Oligos (F5) | 2.32 | ref^[^[^34^](#_ENREF_34)^]^ |
| Immobilized DNA- pseudo peroxidase Mb (MbD_1_) | 0.014 | ref^[^[^38^](#_ENREF_38)^]^ |
| Immobilized DNA- pseudo peroxidase Mb (MbD_2_) | 0.098 | ref^[^[^38^](#_ENREF_38)^]^ |
| Horseradish peroxidase (HRP) | 0.26 | ref^[^[^39^](#_ENREF_39)^]^ |
| Immobilized DNA-HRPD_1_ | 0.30 | ref^[^[^38^](#_ENREF_38)^]^ |
| DNA-containing-(HRPD_1_+cD) | 1.12 | ref^[^[^38^](#_ENREF_38)^]^ |
| DNA-containing-HRPD_1_ | 2.2 | ref^[^[^38^](#_ENREF_38)^]^ |
| Reconstituted Horseradish peroxidase (rHRP) | 7.9 | ref^[^[^38^](#_ENREF_38)^]^ |
| Native Horseradish peroxidase (nHRP) | 8 | ref^[^[^38^](#_ENREF_38)^]^ |
| WT Tel-4G GQ-hemin | 0.55 | This work |
| GQ-11oxoG-hemin | 7.31 | This work |

## **The estimation of GQ-11C-hemin’s catalytic properties**

To estimate the single-molecule catalytic properties of the GQ-11C-hemin complex, we first determined the relationship between the bulk and single-molecule measurements using GQ-hemin constructs with known activities. The initial velocity *V*_0_ follows the Michaelis–Menten equation,^[^[^40^](#_ENREF_40)^]^

$V_{0}=\frac{V_{\max}\left[ S \right]}{K_{M}+\left[ S \right]}$ (Equation S9),

where *V*_MAX_ is the maximum reaction velocity, [*S*] is the substrate concentration, and *K*_M_ is the Michaelis constant.

*V*_MAX_ relates to the single-molecule reaction rate constant (*k*_cat_) through the following equation^[^[^40^](#_ENREF_40)^]^:

$V_{\max}=k_{\text{cat}}\left[ E \right]$ (Equation S10),

where [*E*] denotes the enzyme concentration, which is the same for all bulk experiments (Figure 1B, 0.25 µM). Therefore, under these conditions, $V_{\max}\propto k_{\text{cat}}$.

For WT Tel-4G GQ-hemin (*K*_M_ = 0.111 ± 0.039 µM) and GQ-11oxoG-hemin (*K*_M_ = 0.026 ± 0.004 µM) (Figure 2F), the *K*_M_ values are much smaller than the substrate concentration used in bulk assays ([*S*] = 10 µM AR). Therefore, the initial velocity can be simplified to:

$$V_{0}=\frac{V_{\max}\left[ S \right]}{K_{M}+\left[ S \right]}\approx V_{\max}\propto k_{\text{cat}},$$

which can be expressed as $V_{0}=a\text{ }k_{\text{cat}}$ (Equation S11), where *a* is a constant. This provides a linear relationship between the initial velocity (*V*_0_) and the single-molecule reaction rate (*k*_cat_).

For WT Tel-4G GQ-hemin, the initial velocity is *V*_0_ = 1.17 a.u./min (Figure 1 & Table S2), and the single-molecule reaction rate is *nk*_cat_= *k*_cat_= 0.06 s^-1^ (Figure 2F) given that each GQ-hemin complex contains a single active site (*n*=1). For GQ-11oxoG-hemin, *V*_0_ = 2.28 a.u./min (Figure 1 & Table S2) and *nk*_cat_= *k*_cat_= 0.19 s^-1^ (Figure 2F). Based on the Equation S11, these values give the following two proportionality relationships:

1.17 (a.u./min) = *a*_WT_*0.06 (s^-1^); 2.28 (a.u./min) = *a*_oxoG_*0.19 (s^-1^),

from which we obtain,

*a*_WT_ = 0.325 (a.u.), *a*_oxoG_ = 0.2 (a.u.).

The average proportionality constant is therefore,

*a*_average_ = (*a*_WT_ ^+^ *a*_oxoG_)/2 = 0.263 (a.u.).

To estimate the optimized single-molecule reaction rate of GQ-11C-hemin, we use its initial velocity at the optimized mechanical stress (Figure 4D, 8.70 mW/cm^2^ power):

*V*_0_ = 3.99 (a.u./min) = 0.0665 (a.u./s) = *a*_average_ * *k*_cat_ (s^-1^) = 0.263 (a.u.) * *k*_cat_ (s^-1^),

which gives,

*k*_cat_ = 0.253 (s^-1^).

Assuming the *K*_M_ of the GQ-11C-hemin can be approximated by that of GQ-11oxoG-hemin (0.026 µM, Figure 2F), the catalytic efficiency of the GQ-11C-hemin at optimized mechanical stress (8.70 mW/cm^2^ power, Figure 4D) is:

$\frac{nk_{cat}}{K_{M}}$ = 1*0.253(s^-1^)/(0.026 (μM)) = 9.73 (s⁻¹ μM⁻¹).

## **Table S8.** Estimation of the GQ-11C-hemin catalytic efficiency at optimized mechanical stress of 8.70 mW/cm^2^ power (see Figure 4).

| **Sample** | **Bulk catalytic activity** | **Catalytic efficiency** |
| --- | --- | --- |
| WT Tel-4G GQ-hemin | 1.17 ((a.u.)/min) [Figure 1C]  no ultrasound | 0.548 (s^−1^μM^−1^) [Figure 2F] |
| GQ-11oxoG-hemin | 2.28 ((a.u.)/min) [Figure 1C]  no ultrasound | 7.31 (s^−1^μM^−1^) [Figure 2F] |
| GQ-11C-hemin | 3.99 ((a.u.)/min) [Figure 4D]  at 8.70 mW/cm^2^ ultrasound | ~9.73 (s^−1^μM^−1^) [estimated] |
|  |  |  |

## S11. References

[1] D. Qiu, M. Cheng, P. Stadlbauer, J. Chen, M. Langer, X. Zhang, Q. Gao, H. Ju, J. Sponer, J.-L. Mergny. Topology of DNA G-quadruplexes can be harnessed in holliday junction-based DNA suprastructures to control and optimize their biocatalytic properties. *ACS Catal.* **2023**, *13* (16), 10722, https://doi.org/10.1021/acscatal.3c02818.

[2] J. L. Mergny, L. Lacroix. Analysis of thermal melting curves. *Oligonucleotides* **2003**, *13*, 515, https://doi.org/10.1089/154545703322860825.

[3] L. A. Marky, K. J. Breslauer. Calculating Thermodynamic Data for Transitions of any Molecularity from Equilibrium Melting Curves. *Biopolymers* **1987**, *26*, 1601, https://doi.org/10.1002/bip.360260911.

[4] L. Zuo, J. Ji, P. Pokhrel, B. Pokhrel, K. Ren, H. Mao, H. Shen. Mechano-Electron Spin Coupling Modulates the Reactivity of Individual Coronazymes. *ChemRxiv* **2023**, https://doi.org/10.26434/chemrxiv-2023-tt4bh.

[5] J. Ji, L. Zuo, B. Pokhrel, P. Pokhrel, S. Shakya, H. Shen, H. Mao. Decoupling Activity and Specificity in Coronazymes. *Small* **2025**, 2500783, https://doi.org/10.1002/smll.202500783.

[6] J. Tang, K. Y. Han. Extended field-of-view single-molecule imaging by highly inclined swept illumination. *Optica* **2018**, *5* (9), 1063, https://doi.org/10.1364/optica.5.001063.

[7] M. Tokunaga, N. Imamoto, K. Sakata-Sogawa. Highly inclined thin illumination enables clear single-molecule imaging in cells. *Nat. Methods* **2008**, *5* (2), 159, https://doi.org/10.1038/nmeth1171.

[8] Z. Yu, D. Dulin, J. Cnossen, M. Kober, M. M. van Oene, O. Ordu, B. A. Berghuis, T. Hensgens, J. Lipfert, N. H. Dekker. A force calibration standard for magnetic tweezers. *Rev. Sci. Instrum.* **2014**, *85* (12), 4904148.

[9] B. Bintu, L. J. Mateo, J.-H. Su, N. A. Sinnott-Armstrong, M. Parker, S. Kinrot, K. Yamaya, A. N. Boettiger, X. Zhuang. Super-resolution chromatin tracing reveals domains and cooperative interactions in single cells. *Science* **2018**, *362* (6413), eaau1783, https://doi.org/10.1126/science.aau1783.

[10] M. Ovesný, P. Křížek, J. Borkovec, Z. Švindrych, G. M. Hagen. ThunderSTORM: a comprehensive ImageJ plug-in for PALM and STORM data analysis and super-resolution imaging. *Bioinformatics* **2014**, *30* (16), 2389, https://doi.org/10.1093/bioinformatics/btu202.

[11] L. Zuo, K. Ren, X. Guo, P. Pokhrel, B. Pokhrel, M. A. Hossain, Z.-X. Chen, H. Mao, H. Shen. Amalgamation of DNAzymes and Nanozymes in a Coronazyme. *J. Am. Chem. Soc.* **2023**, *145* (10), 5750, https://doi.org/10.1021/jacs.2c12367.

[12] W. Xu, J. S. Kong, P. Chen. Single-molecule kinetic theory of heterogeneous and enzyme catalysis. *J. Phys. Chem. C* **2009**, *113* (6), 2393, https://doi.org/10.1021/jp808240c.

[13] H. Mao, P. Luchette. An integrated laser-tweezers instrument for microanalysis of individual protein aggregates. *Sens. Actuators, B* **2008**, *129*, 764, https://doi.org/10.1016/j.snb.2007.09.052.

[14] S. Pandey, Y. Xiang, D. Friedrich, Y. Leng, H. Mao. Direct Measurement of Intermolecular Mechanical Force for Nonspecific Interactions between Small Molecules. *J. Phys. Chem. Lett.* **2021**, *12* (46), 11316, https://doi.org/10.1021/acs.jpclett.1c03142.

[15] S. Pandey, S. Mandal, M. B. Danielsen, A. Brown, C. Hu, N. J. Christensen, A. V. Kulakova, S. Song, T. Brown, K. J. Jensen, J. Wengel, C. Lou, H. Mao. Chirality transmission in macromolecular domains. *Nat. Commun.* **2022**, *13* (1), 76, https://doi.org/10.1038/s41467-021-27708-4.

[16] Z. Yu, D. Koirala, Y. Cui, L. F. Easterling, Y. Zhao, H. Mao. Click Chemistry Assisted Single-Molecule Fingerprinting Reveals a 3D Biomolecular Folding Funnel. *J. Am. Chem. Soc.* **2012**, *134* (30), 12338, https://doi.org/10.1021/ja303218s.

[17] D. Koirala, S. Dhakal, B. Ashbridge, Y. Sannohe, R. Rodriguez, H. Sugiyama, S. Balasubramanian, H. Mao. A Single-Molecule Platform for Investigation of Interactions between G-quadruplexes and Small-Molecule Ligands. *Nat. Chem.* **2011**, *3*, 782, https://doi.org/10.1038/nchem.1126.

[18] D. Koirala, C. Ghimire, C. Bohrer, Y. Sannohe, H. Sugiyama, H. Mao. Long-Loop G-Quadruplexes Are Misfolded Population Minorities with Fast Transition Kinetics in Human Telomeric Sequences. *J. Am. Chem. Soc.* **2013**, *135*, 2235, https://doi.org/10.1021/ja309668t.

[19] J. Ji, D. Karna, H. Mao. DNA origami nano-mechanics. *Chem. Soc. Rev.* **2021**, *50*, 11966, https://doi.org/10.1039/d1cs00250c.

[20] U. Bockelmann, P. Thomen, B. Essevaz-Roulet, V. Viasnoff, F. Heslot. Unzipping DNA with Optical Tweezers: High Sequence Sensitivity and Force Flips. *Biophys. J.* **2002**, *82*, 1537, https://doi.org/10.1016/s0006-3495(02)75506-9.

[21] J. Stigler, F. Ziegler, A. Gieseke, J. C. M. Gebhardt, M. Rief. The Complex Folding Network of Single Calmodulin Molecules. *Science* **2011**, *334* (6055), 512, https://doi.org/10.1126/science.1207598.

[22] C. Ghimire, S. Park, K. Iida, P. Yangyuoru, H. Otomo, Z. Yu, K. Nagasawa, H. Sugiyama, H. Mao. Direct Quantification of Loop Interaction and π–π Stacking for G-Quadruplex Stability at the Submolecular Level. *J. Am. Chem. Soc.* **2014**, *136* (44), 15537, https://doi.org/10.1021/ja503585h.

[23] Z. Yu, V. Gaerig, Y. Cui, H. Kang, V. Gokhale, Y. Zhao, L. H. Hurley, H. Mao. Tertiary DNA Structure in the Single-Stranded hTERT Promoter Fragment Unfolds and Refolds by Parallel Pathways via Cooperative or Sequential Events. *J. Am. Chem. Soc.* **2012**, *134* (11), 5157, https://doi.org/10.1021/ja210399h.

[24] J. Ji, A. Sharma, P. Pokhrel, D. Karna, S. Pandey, Y.-R. Zheng, H. Mao. Dynamic Structures and Fast Transition Kinetics of Oxidized G-Quadruplexes. *Small* **2024**, *20* (35), 2400485, https://doi.org/10.1002/smll.202400485.

[25] Z. A. Waller, S. A. Sewitz, S.-T. D. Hsu, S. Balasubramanian. A small molecule that disrupts G-quadruplex DNA structure and enhances gene expression. *J. Am. Chem. Soc.* **2009**, *131* (35), 12628, https://doi.org/10.1021/ja901892u.

[26] M. T. Woodside, W. M. Behnke-Parks, K. Larizadeh, K. Travers, D. Herschlag, S. M. Block. Nanomechanical measurements of the sequence-dependent folding landscapes of single nucleic acid hairpins. *Proc. Natl. Acad. Sci. U S A* **2006**, *103* (16), 6190, https://doi.org/10.1073/pnas.0511048103.

[27] T. A. Laurence, X. Kong, M. Jager, S. Weiss. Probing structural heterogeneities and fluctuations of nucleic acids and denatured proteins. *Proc. Nat. Acad. Sci. USA* **2005**, *102*, 17348, https://doi.org/10.1073/pnas.0508584102.

[28] J. B. Mills, E. Vacano, P. J. Hagerman. Flexibility of single-stranded DNA: use of gapped duplex helices to determine the persistence lengths of poly(dT) and poly(dA). *J. Mol. Biol.* **1999**, *285*, 245, https://doi.org/10.1006/jmbi.1998.2287.

[29] J. Dai, C. Punchihewa, A. Ambrus, D. Chen, R. A. Jones, D. Yang. Structure of the intramolecular human telomeric G-quadruplex in potassium solution: a novel adenine triple formation. *Nucleic Acids Res.* **2007**, *35* (7), 2440.

[30] Y. Wang, D. J. Patel. Solution structure of the human telomeric repeat d[AG3(T2AG3)3] G-tetraplex. *Structure* **1993**, *1* (4), 263, https://doi.org/10.1016/0969-2126(93)90015-9.

[31] P. Pokhrel, G. Sharma, J. Jenyk, A. Lower, J. Ji, S. Shakya, J. Haun, H. Mao. Modulating a Massive Set of Biomolecular Structures by Sono-Mechanical Force. *Adv. Sci.* **2025**, e11687, https://doi.org/10.1002/advs.202511687.

[32] T. Leighton. The Acoustic Bubble. *Academic Press: London* **1994**, 234, https://doi.org/10.1016/B978-0-12-441920-9.X5001-9.

[33] J. Mohanty, N. Barooah, V. Dhamodharan, S. Harikrishna, P. Pradeepkumar, A. C. Bhasikuttan. Thioflavin T as an efficient inducer and selective fluorescent sensor for the human telomeric G-quadruplex DNA. *J. Am. Chem. Soc.* **2013**, *135* (1), 367, https://doi.org/10.1021/ja309588h.

[34] M. Glettenberg, C. M. Niemeyer. Tuning of peroxidase activity by covalently tethered DNA oligonucleotides. *Bioconjugate Chem.* **2009**, *20* (5), 969, https://doi.org/10.1021/bc800558g.

[35] Y. Xiao, J. Hong, X. Wang, T. Chen, T. Hyeon, W. Xu. Revealing kinetics of two-electron oxygen reduction reaction at single-molecule level. *J. Am. Chem. Soc.* **2020**, *142* (30), 13201, https://doi.org/10.1021/jacs.0c06020.

[36] K. S. Han, G. Liu, X. Zhou, R. E. Medina, P. Chen. How does a single Pt nanocatalyst behave in two different reactions? A single-molecule study. *Nano Lett.* **2012**, *12* (3), 1253, https://doi.org/10.1021/nl203677b.

[37] B. Dong, Y. Pei, F. Zhao, T. W. Goh, Z. Qi, C. Xiao, K. Chen, W. Huang, N. Fang. In situ quantitative single-molecule study of dynamic catalytic processes in nanoconfinement. *Nat. Catal.* **2018**, *1* (2), 135, https://doi.org/10.1038/s41929-017-0021-1.

[38] L. Fruk, J. Müller, C. M. Niemeyer. Kinetic analysis of semisynthetic peroxidase enzymes containing a covalent DNA–heme adduct as the cofactor. *Chem. Eur. J.* **2006**, *12* (28), 7448, https://doi.org/10.1002/chem.200501613.

[39] H. H. Gorris, D. R. Walt. Mechanistic aspects of horseradish peroxidase elucidated through single-molecule studies. *J. Am. Chem. Soc.* **2009**, *131* (17), 6277, https://doi.org/10.1021/ja9008858.

[40] A. Cornish-Bowden, H. Dixon, K. Laidler, I. Segel, J. Ricard, S. Velick, E. Webb. Symbolism and terminology in enzyme kinetics. *Eur. J. Biochem* **1982**, *128*, 281, https://doi.org/10.1111/j.1432-1033.1982.tb06963.x.
